# Approximate Bayesian inference in a model for self-generated gradient collective cell movement

**DOI:** 10.1007/s00180-025-01606-5

**Published:** 2025-03-08

**Authors:** Jon Devlin, Agnieszka Borowska, Dirk Husmeier, John Mackenzie

**Affiliations:** 1 Department of Mathematics and Statistics, University of Strathclyde, Glasgow, UK; 2 School of Mathematics and Statistics, University of Glasgow, Glasgow, UK

**Keywords:** Approximate Bayesian computation, Model calibration, Drift-diffusion model, Stochastic differential equations, Chemotaxis

## Abstract

In this article we explore parameter inference in a novel hybrid discrete-continuum model describing the movement of a population of cells in response to a self-generated chemotactic gradient. The model employs a drift-diffusion stochastic process, rendering likelihood-based inference methods impractical. Consequently, we consider approximate Bayesian computation (ABC) methods, which have gained popularity for models with intractable or computationally expensive likelihoods. ABC involves simulating from the generative model, using parameters from generated observations that are “close enough” to the true data to approximate the posterior distribution. Given the plethora of existing ABC methods, selecting the most suitable one for a specific problem can be challenging. To address this, we employ a simple drift-diffusion stochastic differential equation (SDE) as a benchmark problem. This allows us to assess the accuracy of popular ABC algorithms under known configurations. We also evaluate the bias between ABC-posteriors and the exact posterior for the basic SDE model, where the posterior distribution is tractable. The top-performing ABC algorithms are subsequently applied to the proposed cell movement model to infer its key parameters. This study not only contributes to understanding cell movement but also sheds light on the comparative efficiency of different ABC algorithms in a well-defined context.

## Introduction

Collective cell movement is an essential component of several important biological processes such as wound healing (Li et al. 2013), collective cell migration in embryonic development (Scarpa and Mayor 2016), the movement of leukocytes (white blood cells) to infections in immune response (De Oliveira et al. 2016) and cancer metastasis (Stuelten et al. 2018). Most of these processes depend on a type of collective cell migration known as chemotaxis, the movement of cells along chemical gradients in response to a chemical stimulus. For example, it is well established that chemotaxis plays a key role in cancer metastasis (Roussos et al. 2011). Despite the obvious importance of chemotaxis, the sources of chemoattractants, and how these chemical gradients evolve in response to their depletion from cells, are often unknown (Tweedy et al. 2016).

Biophysical models have become an important and often essential tool in understanding complex biological processes, evidenced by the abundance of models in the literature (Tomlin and Axelrod 2007; Motta and Pappalardo 2013; Hori et al. 2021). These models can be used to help interpret experimental data and better understand the mechanisms underlying the observations. They can also be used to formulate hypotheses, make predictions under perturbations and allow certain aspects of the model to be added or removed to see its effect on the overall process, all of which can then be verified experimentally. We concentrate on quantitative models; those which describe and interpret results by linking mathematical models to quantitative data. There are many different types of quantitative models used within biology. For example, hybrid discrete-continuum models aim to combine different mathematical modelling approaches to try and account for often complicated biological behaviours (Osborne et al. 2010; Spill et al. 2015; Harrison and Yates 2016; Bardini et al. 2017). Whole-cell modelling aims to understand the inner working of cells by accounting for every gene and molecule within a cell (Purcell et al. 2013; Babtie and Stumpf 2017; Bhat and Balaji 2020). These models are often very high-dimensional and computationally expensive but very realistically capture the mechanisms underlying collective cell behaviour. In this paper, we consider using stochastic differential equations (SDEs) to model collective cell movement, an approach explored in a number of previous works (Hu et al. 2010; Shi et al. 2013; Tang et al. 2014; Giurghita and Husmeier 2018). SDEs can be used to describe the migration of individual cells, similar to individual-based models. SDE models can also be used to describe collective migration but work better for small population sizes. When the population size is taken much larger, SDE models can become computationally expensive and so partial differential equation (PDE) models are more suitable in that case.

Using biophysical models with physiologically relevant parameter values with the aim of replicating the results of an experiment is often called the forward problem. Equally important is the opposite: being able to estimate parameter values of a model from experimental data. This is known as the inverse problem or statistical inference, and it has a history of being used for biological problems (Wilkinson 2007; Secrier et al. 2009; Lillacci and Khammash 2010; Pullen and Morris 2014). However, statistical inference is seldom done in cell biology due to the complexity of the models and availability of the data. To the best of our knowledge, Ferguson et al. (Ferguson et al. 2016, 2017) were the first and only attempt at parameter inference for a PDE model describing self-generated gradient chemotaxis (Tweedy et al. 2016). These authors estimated the parameters of their PDE models of collective movement by numerical optimization with bootstrap (Ferguson et al. 2016) and Markov chain Monte Carlo (MCMC) (Ferguson et al. 2017). A related work is that of Devlin et al. (Devlin et al. 2019), who inferred drift and diffusion coefficients in a SDE model of a particle undergoing a directed random walk in the presence of static localization error. Their approach makes heavy use of specific analytical results and fits weighted least-squares to mean-square displacement (MSD) data.

There are three main contributions of this paper. First, we propose a novel, hybrid discrete-continuum model of a population of cells moving in response to a self-generated chemotactic gradient, as motivated by the experimental set-up in Tweedy et al. (Tweedy et al. 2016). To our knowledge, no one has used a drift-diffusion stochastic model to describe self-generated gradient chemotaxis. As the model is complex enough to render likelihood-based inference methods infeasible, our second contribution is to demonstrate how the class of approximate Bayesian computation (ABC) methods can be used to infer key parameters of interest. Our third contribution relates to the problem of algorithm selection. After testing the accuracy of the ABC methods on a related but much simpler drift-diffusion SDE (where the posterior is available in closed form), we compared the best methods from this study to inferring key parameters from our hybrid discrete-continuum model. Among the compared ABC methods, we considered an enhanced two-stage “residual" approach that, to the best of our knowledge, has not been used in an ABC setting. We note that ABC has been applied to SDE models before (Picchini 2014; Sun et al. 2015; Zhu et al. 2016; Picchini and Samson 2018; Picchini and Forman 2016; Kypraios et al. 2017; Maybank et al. 2017; Buckwar et al. 2020).

The structure of this paper is as follows. We present a new cell movement model in Sect. 2 and illustrate its ability to simulate self-generated cell chemotaxis. In the same section we also discuss the tractable toy problem based on the drift-diffusion dynamics as well as the mean-square displacement, which is a popular tool for analysing trajectories from SDEs and which we will use to form summary statistics for ABC. In Sect. 3 we revise popular ABC algorithms, where we also describe two enhanced algorithms. We discuss the ABC comparison results for the toy problem in Sect. 4. Results for parameter estimation using ABC for the cell movement model are reported in Sect. 5. Section 6 concludes with a discussion.

## Model for self-generated gradient cell chemotaxis

**Fig. 1:**
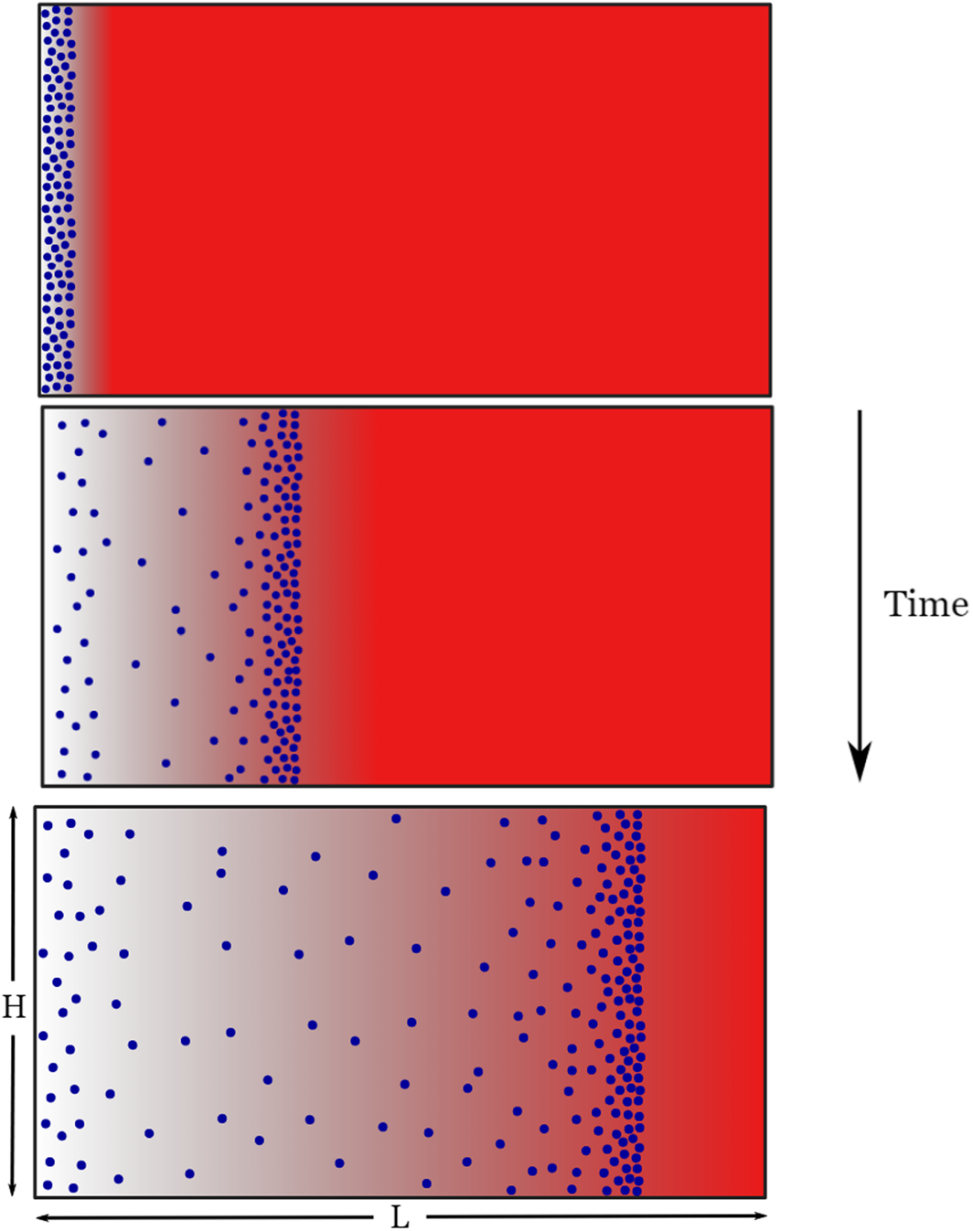
Illustration of the experimental set-up: the blue circles represent cells and the red areas represent the chemical attractant

Motivation for the development of a model of self-generated gradient chemotaxis comes from the experiment of Tweedy et al. (2016). *Dictyostelium discoideum* cells move within a two dimensional chamber of length *L* and height *H*. Initially, a saturating level of the chemoattractant, folic acid, is uniformly dissolved in an agarose gel. As the cells are introduced into a small well at the left hand side of the chamber it is observed that they gradually migrate away from the well by creating a self-generated gradient of the chemoattractant as depicted in Fig. 1. An analysis of cell migration data from (Tweedy et al. 2016) using Kolmogorov-Smirnov tests confirmed that the cell coordinates in the *y*-direction were not significantly different from samples from uniform distributions, indicating that there are no interesting features to be explained in the *y*-direction (Ferguson et al. 2017). To allow for efficient parameter inference, in the following subsections we therefore present a one-dimensional model for self-generated chemotaxis.

### Model of the discrete cell movement

We model the movement of each individual cell by the one-dimensional drift-diffusion SDE

1 $$\begin{aligned} \textrm{d}X_{t}= \nu \, \textrm{d}l \, \frac{K_d}{(K_d+c)^2} \, \frac{\partial c}{\partial x}\,\textrm{d}t+\sqrt{2D}\,\textrm{d}W_{t}, \end{aligned}$$

where $X_{t}$ is the location of the cell at time *t*, $\nu$ is a parameter which converts the difference in receptor occupancy across a cell diameter to a cell velocity due to chemotaxis, $\textrm{d}l$ is the diameter of the cell, $K_d$ is the disassociation constant describing the interaction between the chemotactic ligand and its membrane bound receptor, $c\equiv c(x,t)$ is the concentration of the chemical at position *x* at time *t*, *D* is a measure of the random motion of the cells, assumed to be equal and constant for all cells, and $W_{t}$ is a Wiener process. Here, the domain of $X_{t}$ is [0, *L*], with the initial condition $X_{0}=\upsilon$, where $\upsilon$ follows the uniform distribution *U*(0, *L*/20). Note that this uniform distributions ensures that the cells begin in a small well of length $L/20 \, \mu \textrm{m}$. To ensure that the cells remain in the chamber, we impose the boundary conditions $X_{t}=0$, if at any stage $X_{t}<0$, and $X_{t}=L$, if any cell is predicted to have $X_{t}>L$.

The chemotaxis velocity term in (1) is motivated by looking at receptor-ligand kinetics. First, imagine cells interacting with a chemical attractant. Over time, ligands begin to bind on and off the cell receptors. The rate at which ligands bind on to the receptors depends on the number of free receptors and the concentration of the chemical, while the rate at which they bind off the receptors depends on the number of bound receptors. From this, if we let $\psi$ denote the number of bound receptors, then we have

2 $$\begin{aligned} \frac{\partial \psi }{\partial t}=k_1c(R_{tot}-\psi )-k_{-1}\psi , \end{aligned}$$

where $k_1,k_{-1}$ are the rates at which the ligand binds on and off the receptors, respectively, and $R_{tot}$ is the total receptor number. For simplicity, we assume that $R_{tot}$ is constant. Denoting $R=\psi /R_{tot}$ as the fractional receptor occupancy, we can rewrite (2) so that

3 $$\begin{aligned} \frac{\partial R}{\partial t}&= (k_1c+k_{-1})\left( \frac{k_1c}{k_1c+k_{-1}}-R\right) . \end{aligned}$$

If the chemical concentration remains constant over a long enough time scale, then we can assume that the receptor occupancy reaches an equilibrium value where ${\partial R}/{\partial t}=0$. Therefore, we get

4 $$\begin{aligned} R=\frac{c}{K_d+c}, \end{aligned}$$

where $K_d=k_{-1}/k_1$ denotes the disassociation constant. From (4) we can see that the disassociation constant is the ligand concentration which results in half the total number of receptors being occupied. It is easy showed that equation (2) has solution

5 $$\begin{aligned} \psi =\frac{k_1 R_{tot}c}{k_1 c+k_{-1}}+\left( \psi _0-\frac{k_1 R_{tot}c}{k_1 c+k_{-1}}\right) \exp (-(k_1 c+k_{-1})t), \end{aligned}$$

where $\psi _0$ is the initial number of bound receptors. We can see therefore that the rate to reach equilibrium is determined by $k_1 c+k_{-1}$. We will assume that the initial background concentration $c\gg K_d$, and so

6 $$\begin{aligned} k_1c+k_{-1}=(k_1c-k_{-1})+2k_{-1}>k_1c-k_{-1}\gg 0. \end{aligned}$$

Therefore, the exponential term in (5) will decay rapidly, and so the timescale to reach equilibrium will be small compared to the other processes taking place (further justification about this assumption is given in Appendix A).

Denoting the difference in fractional receptor occupancy from the front to the back of the cell by $\Delta R$, we can approximate this by

7 $$\begin{aligned} \Delta R&\approx \textrm{d}l \, \frac{K_d}{(K_d+c)^2} \, \frac{\partial c}{\partial x}. \end{aligned}$$

If we assume that the chemotactic velocity is proportional to $\Delta R$ with velocity $\nu$, then we arrive at (1). The chemotactic term in (7) is similar to that used in Hillen and Painter (2009) and others (Segel 1977; Tyson et al. 1999). They looked at PDE chemotaxis models of advection–diffusion type, where the advection models the cell density movement.

It is instructive to consider the behaviour of this chemotatic term under different scenarios. For example, if we have a steady state relative concentration gradient, then

$$\begin{aligned} \frac{{\Delta c}}{c_{0}} \approx \frac{\textrm{d}l}{c_{0}}\frac{\partial c}{\partial x} = \textrm{constant}, \end{aligned}$$

where ${\Delta c}$ and $c_{0}$ denotes the difference and average concentration across the cell, respectively. In this situation we find that

8 $$\begin{aligned} \Delta R \propto \frac{c K_{d}}{(K_{d}+c)^{2}}. \end{aligned}$$

We can see that the chemotactic term therefore decays to zero as the absolute concentration level tends to zero as expected. We also see that $\Delta R\rightarrow 0$ when $c\gg K_{d}$, as in this situation almost all of the cell’s receptors are occupied and hence it is difficult for the cell to determine the gradient of the chemoattractant. It is easy to show that in fact $\Delta R$ is maximised when $c \approx K_{d}$. At this level of chemoattractant, roughly half of the cell’s receptors are occupied at the front and the back of the cell.

### Model of the continuous chemical concentration

We assume that the chemical concentration evolves according to a constant coefficient diffusion equation with moving point sinks to model the degradation of the chemical by membrane-bound enzymes on each cell. The governing equation is therefore

9 $$\begin{aligned} \frac{\partial c}{\partial t}= & D_{c}\frac{\partial ^2 c}{\partial x^2}-\frac{1}{\sqrt{2\pi \sigma ^2}}\sum _{j=1}^{N_S} \gamma (c(x^{\; (j)},t)) \, \exp \left( \frac{-(x-x^{\; (j)})^2}{2\sigma ^2}\right) , \end{aligned}$$

10 $$\begin{aligned} c(x,0)= & c_0, \quad t>0, \end{aligned}$$

where $D_{c}$ is the diffusion coefficient of the chemical, $x^{\; (j)}$ is the location of the *j*th cell, $\sigma ^2$ is variance of the Gaussian degradation term, $c_0$ is the initial concentration and $\gamma (c(x^{\; (j)},t))$ denotes the rate of decay of the chemical at the *j*th cell. The strength of the cell degradation is modelled using a Michaelis-Menten formulation

11 $$\begin{aligned} \gamma (c(x^{\; (j)},t))=\frac{V_{max} \, c(x^{\; (j)},t)}{K_m+c(x^{\; (j)},t)}, \end{aligned}$$

where $V_{max}$ is the maximum rate of degradation and $K_m$ is the Michaelis-Menten constant.

### Numerical discretisation

We assume that there are $N_S$ cells which are simulated over the time interval $0\le t\le T$. The total time of simulation *T* should be commensurate with the observational time over which experimental data is collected and as such is a possible experimental design parameter. The time interval is assumed to be partitioned uniformly by the *N* time points, $t_{n}=(n-1)T/(N-1)=(n-1)\Delta t$, $n=1, \ldots , N$. The position of the *j*th cell at the *n*th time point is given by $x_{n}^{\;(j)}, \; 1 \le n \le N, \; 1 \le j \le N_S$.

The cells are moved by solving numerically the SDE (1) by the Euler-Maruyama method. This gives

12 $$\begin{aligned} x_{n+1}^{\; (j)}=x_{n}^{\; (j)} + \nu \, \textrm{d}l \frac{K_d}{(K_d+c_{n}^{\; (j)})^2} \, \frac{\partial c_n}{\partial x} \, \Delta t + \sqrt{2D} \, \Delta W_n, \quad 1 \le n \le N, \quad 1 \le j \le N_S, \end{aligned}$$

where $c_{n}^{\; (j)}$ is the chemical concentration evaluated at the location of the *j*th cell at the *n*th time point, $\partial c_n/\partial x$ is the chemical gradient evaluated at the location of the *j*th cell at the *n*th time point, and $\Delta W_n=W_{t_{n+1}}-W_{t_{n}}$ follows a normal distribution of the form $\mathcal{N}(0,\Delta t)$.

Notice that equation (12) depends on the concentration and gradient of the concentration for each cell over all time. To estimate these quantities, we will use an implicit-explicit finite difference scheme to numerically solve (9). To do this, we split the spatial domain into $N_X+1$ points, $x^i=(i-1)L/N_X=(i-1)h$, for $i=1,\ldots ,N_{X}+1$. Then, denoting the approximation of the concentration at the point $x^i$ at time point $t_k$ by $c_{k}^{i}$, we look to solve

13 $$\begin{aligned} \frac{c_{k+1}^{i}-c_{k}^{i}}{\Delta t}=&D_c \left( \frac{c_{k+1}^{i+1}-2c_{k+1}^{i}+c_{k+1}^{i-1}}{h^2}\right) \nonumber \\&-\frac{1}{\sqrt{2\pi \sigma ^2}}\sum _{j=1}^{N_S} \frac{V_{max} \, c(x_{k}^{\; (j)})}{K_m+c(x_{k}^{\; (j)})} \, \exp \left( \frac{-(x^{i}-x_{k}^{\; (j)})^2}{2\sigma ^2}\right) , \end{aligned}$$

for $c_{k+1}^{i}$, along with an approximation of the boundary conditions that $\partial c/\partial x=0$ at $x=0$ and $x=L$ which gives $c_{k+1}^{0}=c_{k+1}^{2}$ and $c_{k+1}^{N_X-1}=c_{k+1}^{N_X+1}$. The updated concentration $c^{i}_{k+1}$, $i=1,\ldots ,N_{X}+1$ can be obtained by solving a tri-diagonal system of equations. Once we have calculated the concentration at the $N_X+1$ spatial points, we use linear interpolation to estimate the concentration at the location of the cells. Similarly, we use a linear approximation of the gradient of the concentration so that $\partial c/\partial x \approx (c_{k+1}^{i+1}-c_{k+1}^{i-1})/2\,h$ when $x=x^i$, and again use linear interpolation to estimate its value at the location of the cells. The same size of time step is used to solve (13) as is used to moved the cells in (12).

Once we have solved numerically equation (12), we must ensure that the cells remain in the simulated chamber by imposing appropriate boundary conditions. This is done by assuming that if $x_{n+1}^{\;(j)}<0$, then $x_{n+1}^{\;(j)}=0$, and if $x_{n+1}^{\;(j)}>L$, then $x_{n+1}^{\;(j)}=L$.

### Self-generated gradient simulations

The dataset of Tweedy et al. (2016) contains the coordinates of a group of *Dictyostelium discoideum* cells moving by self-generated gradients under a plate of agarose of length $L=2500 \, \mu \textrm{m}$. The time taken for the cells to traverse the majority of the plate length is $T=5.5 \, \textrm{h}=19800 \, \textrm{s}$. Initially, there is a uniform amount of folate of concentration $c_0=10 \, \mu \textrm{M}$ that covers the entire chamber.

Our mathematical model (1) and (9) is parameterised by a seven-dimensional vector ${\varvec{\theta }}=(D, K_d, \textrm{d}l, \nu , D_c, V_{max},K_m)^{T}$. When available, the physical parameters in our model are set to literature values, see Table 1, with the following modifications: $V_{max}$ is set to a slightly higher value to allow for the relatively low number of simulated cells ($N_{S}=100$). The diffusion coefficient for folic acid, $D_c$, is based on the estimate in Kalimuthu and John (2009), but slightly reduced to allow for the fact that we do not have diffusion in solution, but in an agarose gel.

As opposed to the other parameters, which refer to physical quantities that can in principle be directly measured, $\nu$ and *D* characterise the collective cell movement and its interaction with the environment. This is a complex system that defies parameter estimation by direct measurement, and we therefore have to infer them based on the observed cell movement itself. The former does not have an equivalent literature value. This value controls how far along the domain the cells will travel. We have therefore chosen a value of $\nu = 31.57 \mu \mathrm{m/s}$, which allows the cells to move a similar distance as those from Tweedy et al. (2016).

Studies of tracks of cell movement in isotropic environments reveals a common feature that cells typically maintain their direction of motion over short time periods, but over longer periods the direction of movement becomes random. This type of motion is normally referred to as a persistent random walk. The short time period where cells maintain their direction is called the directional persistence time. An analysis of the mean squared displacement of a persistent random walk indicates that an estimate for *D* can be obtained from the expression $D=t_{p}v^{2}/2$, where $t_{p}$ is the directional persistence time and *v* is the speed of an individual cell (Dickinson and Tranquillo 1993). Li et al. (2008) carried out careful single-cell experiments on Dictyostelium cells and found $t_{p}=8$ minutes and $v=8 \mu \mathrm{m/minute}$. Therefore, we can estimate that $D\approx 3 \mu \textrm{m}^{2}/s$. A similar value can be deduced from the gradient of a straight line fit to the long time mean-squared-displacement data in Bosgraaf and van Haastert (2009) for Dictyostelium cells migrating in the absence of a chemoattractant.

**Table 1:** Nominal model parameter values for the simulation of *Dictyostelium discoideum* cells moving in response to a self-generated gradient in the chemoattractant folic acid

Parameter	Dimensional	Reference
$K_d$	$150 \, \textrm{nM}$	Wurster and Butz (1980)
$\textrm{d}l$	$10 \mu m$	Rivero et al. (1996)
$D_c$	$11.05 \, \mu \mathrm{m^2/s}$	Kalimuthu and John (2009)$^{*}$
$V_{max}$	$3 \times 10^{-2} \, \mathrm{nM/s}$	Kakebeeke et al. (1980)$^{*}$
$K_m$	$5 \, \mu \textrm{M}$	Kakebeeke et al. (1980)

The asterisk $(^{*})$ indicates that the corresponding reference values from the literature were adjusted as discussed in the main text to match our model

**Fig. 2:**
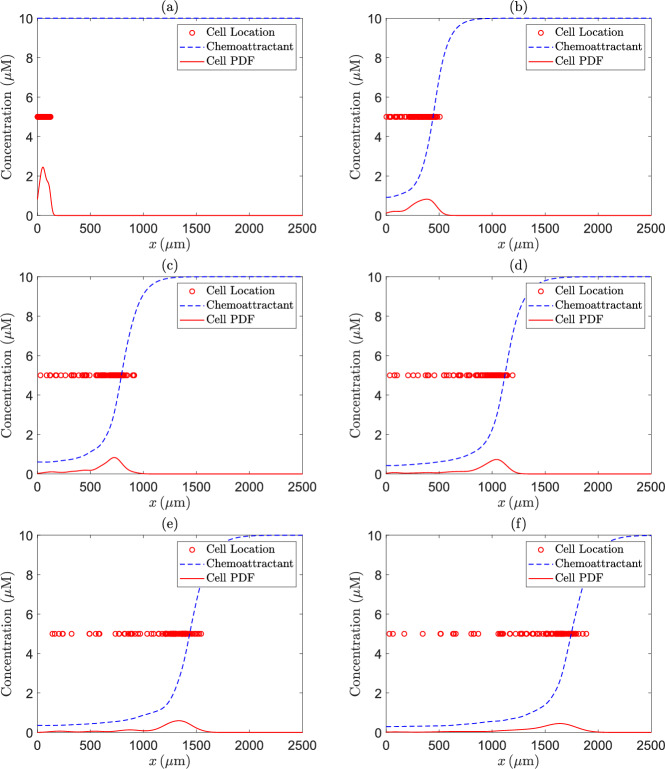
The cell locations (red circles), the chemottractant concentration (dashed blue line) and the cell location PDF (solid red line) over time, where time progresses from (**a**) to (**f**)

We calculate the location of the cells by (12) and the chemical concentration by (13). We take $N_X=1000$, giving a spatial grid size of $h=2.5 \, \mu \textrm{m}$ for the implicit-explicit finite difference scheme. Simulations are performed using $N_{s}=100$ cells and the time interval is discretised using $N=500$ time steps, and hence the time step $\Delta t=19800/499=39.68 \, \textrm{s}$. Initially, the cells are given the position $x_{1}^{\; (j)}=125\upsilon$, where $\upsilon$ follows a standard uniform distribution *U*(0, 1). Note that this condition ensures that the cells begin in the small well. To verify the correctness of the proposed numerical solution and empirically prove convergence, we analyse a progression of the location of the cells, chemical concentration profile and the cell location probability density function (PDF) at six equally spaced time points (see Fig. 2). We can see that the cells move from left to right as expected. We see a leading wave of cells, a key property of self-generated gradient chemotaxis. Tweedy et al. (2016) measure the chemical concentration profile at a single time point corresponding to the end of the experiment. They find that the chemical concentration is high in front of the cell wave and quickly drops off to near zero concentration at the location of the wave. We see very similar results with our simulated concentration profiles. Finally, we find a single mode in the cell location PDF corresponding with the cell wave, whereas the experiments done by Tweedy et al. (2016) find a bimodal distribution for the PDF. In their experiments, new cells continue to move into the chamber during the experiment, while in our simulated experiments, the number of cells in the chamber is constant from the start. We believe this is why we do not find a bimodal cell location PDF.

**Fig. 3:**
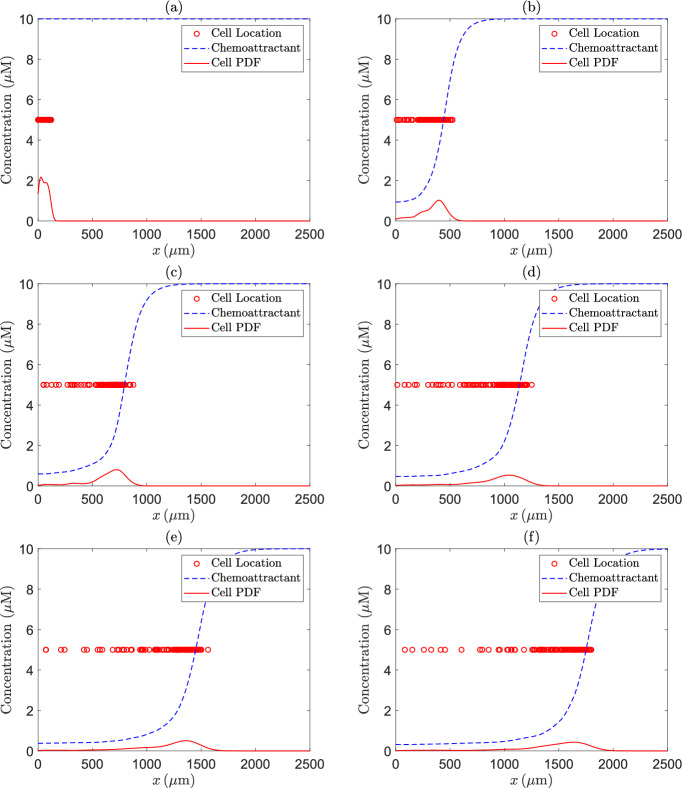
The cell locations (red circles), the chemoattractant concentration (dashed blue line) and the cell location PDF (solid red line) over time, where time progresses from (**a**) to (**f**), for the same parameter values as in Fig. 2, except for the values of *N* and $N_X$, which we doubled to halve both the time step and the spatial grid size

To test whether the time and space steps used in the Euler-Maruyama method and the implicit-explicit finite difference scheme give rise to accurate numerical approximations, we repeat the simulations which led to Fig. 2 with doubled values of *N* and $N_X$ (which results in a halving of both the time step and the spatial grid size). The results shown in Fig. 3 are almost identical to those in Fig. 2, suggesting that the original values for *N* and $N_{X}$ give rise to accurate numerical approximations.

### Toy problem

Below we introduce a simple drift-diffusion SDE, which we refer to as the “toy problem”. The purpose of this model is to facilitate selecting an appropriate ABC algorithm for inference in the proposed cell movement model. Comparing ABC methods directly on the cell movement model would be too computationally involved due to the complexity of that model.

We consider the following one-dimensional drift-diffusion SDE:

14 $$\begin{aligned} \textrm{d}X_{t}=\alpha \,\textrm{d}t+\sqrt{2D}\,\textrm{d}W_{t}, \end{aligned}$$

where $X_{t}\in \mathbb {R}$ denotes the true location of a particle^1^ at time *t*, with the initial condition $X_{0}=0$, and $\textrm{d}W_{t}$ is the increment of a Wiener process. The particles are assumed to move in an infinite domain, so there are no boundary conditions. The model parameters are $\alpha$, the drift velocity, and *D*, the diffusion coefficient, which we collect in ${\varvec{\theta }}=(\alpha ,D)^{T}$. In the simulation study in Sect. 4, for both parameters we adopt the uniform prior distribution from 0 to 10, denoted *U*(0, 10).

#### Numerical solution

Due to its simplicity, model (14) admits an exact solution. However, to make our discussion of selecting an appropriate ABC algorithm (Sect. 4.3.1) general and applicable to more complex SDE models – that do require numerical methods for solving – we present a general solution to (14) based on the Euler-Maruyama method.

We assume ${\varvec{x}}^{(j)}$, the *j*th trajectory generated from (14), $j=1,\dots ,N_{S}$, is measured at *N* time points $t_n=(n-1)T/(N-1)$, $n=1,\ldots ,N$, covering the measurement time range [0, *T*], which we denote ${\varvec{x}}^{(j)}=\{x_{n}^{(j)}\}_{n=1}^{N}$. We set $N=100$ (the number of discretization time points) and $N_S=100$ (the number of generated trajectories).

#### Exact posterior distribution

Under model (14), the likelihood for the *n*th time (measurement) point from the *j*th trajectory at time *t* is given by Codling et al. (2008)

15 $$\begin{aligned} p(x_{n}^{(j)},t\vert \alpha ,D)=\frac{1}{\sqrt{4\pi Dt}}\exp \left( \frac{- (x_{n}^{(j)}-\alpha t)^2}{4Dt}\right) , \end{aligned}$$

which means that the likelihood for the whole trajectory ${\varvec{x}}$ has the following form

16 $$\begin{aligned} L({\varvec{x}}^{(j)}\vert \alpha ,D)= & \prod _{n=2}^{N} p(x_n^{(j)},t \vert x_{n-1}^{(j)},\alpha ,D) \nonumber \\= & (4\pi D\textrm{d}t)^{-\frac{N}{2}}\exp \left( \frac{-\sum _{n=2}^{N} (\Delta x_n^{(j)} -\alpha \Delta t)^2}{4D\Delta t}\right) , \end{aligned}$$

where $\Delta t=T/(N-1)$ is the step size and $\Delta x_n^{(j)} =x_n^{(j)} -x_{n-1}^{(j)}$. We assume that each step from $x_n^{(j)}$ to $x_{n+1}^{(j)}$ is equivalent to taking a time step of size $\Delta t$ starting from $x_n^{(j)}=0$. The likelihood for the population data ${\varvec{y}}=\{{\varvec{x}}^{(1)},\dots ,{\varvec{x}}^{(N_{S})}\}$ of $N_{S}$ independent trajectories is then a product of the likelihoods for individual trajectories

17 $$\begin{aligned} L({\varvec{y}}\vert \alpha ,D)&=\prod _{j=1}^{N_{S}} L({\varvec{x}}^{(j)}\vert \alpha ,D). \end{aligned}$$

Notice that the likelihood is tractable, which combined with uniform priors results in a closed form for the posterior.

### Mean-square displacement

The MSD has been traditionally used to analyse trajectory data (Savin and Doyle 2005; Qian et al. 1991; Saxton and Jacobson 1997; Saxton 1997; Devlin et al. 2019). The MSD measures the spatial extent of a random process based on the deviation of the particle location with respect to a reference location (the 0 origin, in our case). The MSD is defined as

18 $$\begin{aligned} \rho (t) \equiv \mathbb {E}(\vert X_{t} \vert ^{2}) = \int x^2 p(x,t\vert \alpha ,D)dx, \end{aligned}$$

where *p*(*x*, *t*) is the pdf of the particle displacement at time *t* given in (15). For a one-dimensional system (14), the MSD can be derived analytically (Devlin et al. 2019) as

19 $$\begin{aligned} \rho (t)=\alpha ^2 t^2+2Dt. \end{aligned}$$

#### MSD estimation

In practice, we cannot use the theoretical, continuous-time formula (19) for the MSD and hence we need to estimate it based on discrete observations. The most popular method to do this is the time-average overlapping MSD (Michalet 2010), which, for the *j*th trajectory, $j=1,\dots ,N_{S}$, is computed as

20 $$\begin{aligned} \rho _{n}^{(j)} = \frac{1}{N+1-n}\sum _{i=1}^{N+1-n}( x_{i+n}^{(j)} - x_{i}^{(j)})^2, \quad n=1,\dots ,N. \end{aligned}$$

Notice that $\rho _{n}^{(j)}$ is computed for each time lag $n\Delta t$ resulting in *N* values of the MSD per trajectory. To obtain more reliable estimates of the MSD we then average individual MSDs over trajectories to obtain the ensemble time-averaged MSD given by

21 $$\begin{aligned} \rho _{n} = \frac{1}{N_{S}}\sum _{j=1}^{N_{S}}\rho _{n}^{(j)}, \quad n=1,\dots ,N. \end{aligned}$$

**Fig. 4:**
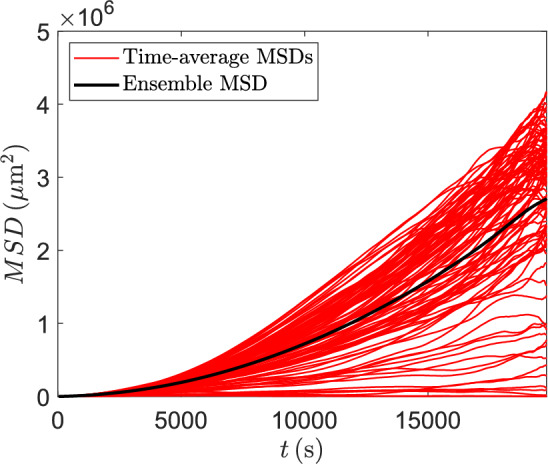
A plot of the time-average overlapping MSDs for each individual cell (red lines) and the ensemble MSD (dashed black line) using the parameter values from Table 1

Figure 4 compares individual MSDs (20) with the ensemble MSD (21); the former are calculated at the $N-1$ non-zero time points for each of $N_S$ cells, with $N=500$ and $N_S=100$ as in Sect. 2.4, and using the parameter values from Table 1. In Sect. 4.3.1 we will use (21) at $N=100$ time points as the summary statistics for comparing the ABC algorithms on the toy problem. Note that using the ensemble time-averaged MSD (21) allows us to limit the number of summary statistics to a pre-selected value even with increasing number of data points as we take the mean of the whole distribution of the MSD values.

#### Properties of the MSD

Formula (19) reveals important properties of the MSD. First, it shows how the two parameters of the SDE model affect the MSD values for different values of *t*. Notice that the MSD is a quadratic function of *t*. For small values of *t* the linear term 2*Dt* dominates, so that the MSD value is mostly determined by the value of *D*, the diffusion coefficient. On the other hand, for large values of *t*, the quadratic term $\alpha ^{2}t^{2}$ prevails, and consequently the value of the drift coefficient $\alpha$ matters most. This will influence the informativeness of the MSD as the chosen summary statistic for inferring $\alpha$ and *D* for different values of *T*. Second, we note that the MSD increases quadratically with *T*, while its variance grows cubically with *T* (Devlin et al. 2019). The latter implies that the estimated MSD becomes less accurate a summary statistics as time increases.

## Approximate Bayesian computation

In an inverse or statistical inference problem we are interested in the posterior distribution $\pi ({\varvec{\theta }} \vert {\varvec{y}})$ of the unknown parameters ${\varvec{\theta }} \in \Theta \subseteq \mathbb {R}^{H}$ of a model given the observed data ${\varvec{y}}$, which by Bayes’ theorem is given as

22 $$\begin{aligned} \pi ({\varvec{\theta }} \vert {\varvec{y}})=\frac{p({\varvec{y}} \vert {\varvec{\theta }})\, \pi ({\varvec{\theta }})}{p({\varvec{y}})}, \end{aligned}$$

where $p({\varvec{y}} \vert {\varvec{\theta }})$ is the likelihood function, $\pi ({\varvec{\theta }})$ is the prior distribution and $p({\varvec{y}})$ is the marginal likelihood. Application of Bayes’ theorem requires computing the likelihood; however, this is not always feasible. For stochastic systems calculation of the likelihood depends on the solution of path integrals over all realisations of the latent state, which typically is analytically intractable. Likelihood-free methods are a common workaround for systems where the likelihood function is not available. The two common likelihood-free approaches are density estimation methods, which approximate the likelihood function numerically, e.g. the synthetic likelihood method (Wood 2010), and ABC, which compares observed and simulated data, or statistics of the data, through use of a distance measure. ABC has gained a considerable interest in recent years and has been used for parameter inference in a wide range of disciplines, from the biological sciences (Pritchard et al. 1999; Beaumont et al. 2002; Lintusaari et al. 2017; Lambert et al. 2018), through image analysis (Moores et al. 2015), epidemiology (Kypraios et al. 2017; McKinley et al. 2018), up to time series analysis (Toni et al. 2009; Drovandi et al. 2016; Martin et al. 2019; Tancredi 2019). We refer to Sisson et al. (2018) for a detailed treatment of ABC.

Below, we first discuss basic rejection ABC (Subsection 3.1). We then move to more advanced schemes, i.e. sequential Monte Carlo (SMC) ABC (Sisson et al. 2007; Prangle 2017) (Sect. 3.2) and semi-automatic ABC (Fearnhead and Prangle 2012) (Sect. 3.3). Finally, in Sect. 3.4 we discuss two novel ABC algorithms, based on standard techniques from computational statistics and machine learning, aimed at mitigating the problems faced by the previous semi-automatic ABC scheme. We present listings of the discussed algorithms in Appendix B.

### Rejection ABC

In the basic modern rejection ABC algorithm (Tavaré et al. (1997), Pritchard et al. (1999); see Algorithm 2 in Appendix B) the prior distribution is sampled to obtain draws ${\varvec{\theta }}^{*}$, which are used to simulate from a generative model ${\varvec{y}}^{*}\sim f({\varvec{y}} \vert {\varvec{\theta }}^{*})$. The parameter value ${\varvec{\theta }}^{*}$ is accepted if $\vert \vert S({\varvec{y}}^*)-S({\varvec{y}}) \vert \vert <\varepsilon$, where the norm $\vert \vert \cdot \vert \vert$, the summary statistics $S(\cdot )$, and the tolerance level $\varepsilon$ must be specified. If the summary statistics used are sufficient, then the approximate posterior distribution approaches the exact posterior distributions as $\varepsilon$ tends to zero. Beaumont et al. (2002) introduced smooth weighting to overcome the problem of the $S({\varvec{y}}^{*})$ values being treated equally whenever $\vert \vert S({\varvec{y}}^*)-S({\varvec{y}}) \vert \vert <\varepsilon$, regardless of the exact value of $\vert \vert S({\varvec{y}}^*)-S({\varvec{y}}) \vert \vert$.

Rejection ABC is subject to considerable computational inefficiencies, especially when dealing with high dimensional parameter spaces or continuous data (Lintusaari et al. 2017). It is also inefficient when the prior and posterior distribution are vastly different, requiring small tolerance values to obtain an accurate estimate of the posterior distribution, thereby increasing the computational cost.

### Sequential Monte Carlo ABC

Sisson et al. (2007) propose an ABC method based on sequential Monte Carlo (SMC) with partial rejection control (Liu 2001). Their algorithm samples over a sequence of $N_{\varepsilon }$ intermediary distributions, with decreasing tolerance values $\varepsilon _1, \varepsilon _2, \ldots , \varepsilon _{N_{\varepsilon }}$ leading to closer approximations to $\pi ({\varvec{\theta }}\vert S({\varvec{y}}))$. In each iteration samples are generated from the previously found intermediary distribution, except the initialization for which the prior distribution is used. The generated draws are then perturbed with Markov transition kernels and, to preserve convergence properties, weighted appealing to the importance sampling argument (Douc et al. 2007). The resulting approximation to the posterior is biased (Beaumont et al. 2009) and a number of unbiased alternatives has been proposed (Beaumont et al. 2009; Toni et al. 2009; Beaumont 2010) (a general version of one of them Beaumont 2010 is provided in Appendix B, Algorithm 3)

Sampling from distributions that are progressively becoming closer to the target distribution $\pi (\theta \vert S(y))$ reduces the number of parameter values drawn from low probability regions, thus enhancing computational efficiency compared to the rejection method. However, for low tolerance values, the probability of accepting parameter values can become small, even for good proposal distributions. This may result in the algorithm being ran for longer than needed with little improvement in the inference (Lintusaari et al. 2017).

***Adaptive weighting***

When the summary statistics are not standardised (Pritchard et al. 1999; Beaumont et al. 2002), i.e. they are out of scale with each other, the largest summary statistic can dominate over the others during the acceptance step. To overcome this problem Prangle (2017) proposes an adaptive approach to weighting summary statistics, focusing on the weighted Euclidean distance and adopting the median absolute deviation (MAD) for weighting. During the first ABC-SMC iteration, equal weights are used, while in all later iterations, the weights are based on the MAD of the accepted summary statistics from the previous iteration. The tolerance values are chosen automatically as a quantile of the previous accepted distance values (Drovandi and Pettitt 2011). Algorithm 4 in Appendix B summarises Prangle’s (2017) algorithm.

### Standard semi-automatic ABC

Fearnhead and Prangle (2012) propose a semi-automatic approach to selecting summary statistics, in which the posterior mean serves as the summary statistics. Fearnhead and Prangle (2012) demonstrate that this approach minimizes the quadratic loss measuring the estimation accuracy. The posterior mean is not known a priori and hence in practice it needs to be estimated using a regression model. Below we will refer to semi-automatic summary statistics as “second-stage” summary statistics, to distinguish them from “first-stage” summary statistics that are obtained directly as transformations of the data. Algorithm 1 presents a high-level description of semi-automatic ABC.

**Algorithm 1:**
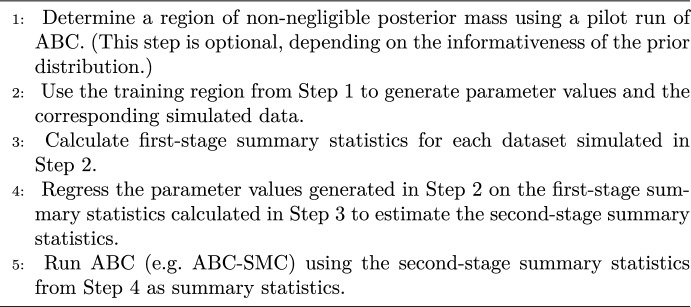
High-level description of semi-automatic ABC.

Note that the regression model in Step 4 can be selected in different ways, which we discuss in Sects. 3.3.1 (multivariate linear regression), 3.3.2 (Gaussian process regression) and 3.4 (two modified approaches).

#### Linear regression approach

The original approach of Fearnhead and Prangle (2012) adopts multivariate linear regression (MVLR) of parameter values on first-stage summary statistics. Formally, suppose we have generated a set of *M* parameter values ${\varvec{\theta }}^{(j)}$ and the corresponding observations ${\varvec{y}}^{(j)}$, $j=1, \ldots , M$ (Step 2 in Algorithm 1). Let $g(\cdot )$ be a vector-valued function representing transformations of the data and $\textbf{z}$ denote a vector of transformations under *g*, $\textbf{z}:=g({\varvec{y}})\in \mathcal {X}\subseteq \mathbb {R}^{G}$. We refer to $\textbf{z}$ as “first-stage summary statistics” (see Step 3 in Algorithm 1). As an illustration, consider an application by Fearnhead and Prangle (2012), who found that $g({\varvec{y}})=[{\varvec{y}}, {\varvec{y}}^2, {\varvec{y}}^3, {\varvec{y}}^4]$, a vector of length $4n_{y}$ (where $n_{y}$ is the length of the data vector ${\varvec{y}}$) consisting of the data together with the second, third and fourth powers of individual data points, resulted in well-performing set of summary statistics. In our applications, $\textbf{z}$ consists of ensemble MSD (21) calculated at either $N=100$ points (in Sect. 4.3.1) or at the first and final ten MSD points (in Sect. 5).

In the semi-automatic ABC approach the posterior mean of the *i*th component of ${\varvec{\theta }}$ is taken as the *i*th “second-stage summary statistic” (see Step 4 in Algorithm 1). Fearnhead and Prangle (2012) propose to estimate that posterior mean by regressing the parameter values $\theta _i$ on the linear of transformations of $\textbf{z}$, i.e.

23 $$\begin{aligned} \theta _i=\mathbb {E}(\theta _i \vert {\varvec{y}})+\epsilon _i=\beta ^{(i)}_0+{\varvec{\beta }}^{(i)} \textbf{z}+\epsilon _i, \end{aligned}$$

where $\epsilon _i$ is a zero-mean noise term. We can use least-squares to fit (23); the fitted value $\hat{\beta }_0^{(i)}+{\varvec{\hat{\beta }}}^{(i)}\textbf{z}$ is an estimate for $\mathbb {E}(\theta _i \vert {\varvec{y}})$.

#### Gaussian process enhanced ABC

Using MVLR in Step 4 of Algorithm 1 is likely to be suboptimal when the relationship between parameters and first-stage summary statistics is nonlinear. More accurate results are then likely to be obtained if we construct second-stage summary statistics using a flexible, nonlinear regression model e.g. using Gaussian processes (GPs) as in Borowska et al. (2021). We refer to this approach as GP-ABC.

GP regression is a nonparametric regression model where the values of the latent function *f* at the corresponding locations $\textbf{z}$ explain the observed values $\textbf{y}$. The unobserved function values are assumed to follow a GP, a stochastic process such that the joint distribution of any finite number of random variables from this process is Gaussian. A GP is fully specified by its mean and covariance function. Rasmussen and Williams (2006) provide an extensive treatment of GPs. Adopting the notation from Sect. 3.3.1, we can write the GP regression model as

24 $$\begin{aligned} \theta _{i} = f_{i}(\textbf{z}) + \eta _{i},\quad \eta _{i} {\mathop {\sim }\limits ^{iid}} \mathcal {N}(\cdot \vert 0,\sigma _{i}^{2}), \end{aligned}$$

where $\mathcal {N}(\cdot \vert \mu ,\sigma ^{2})$ denotes the Gaussian distribution with mean $\mu$ and variance $\sigma ^{2}$ (and *iid* stands for “independently and identically distributed”). The latent values $f_{i}(\textbf{z})$ follow a GP $f_{i}(\textbf{z}) \sim \mathcal{G}\mathcal{P}(m_{i}(\textbf{z}),k_{i}(\textbf{z},\textbf{z}'))$, where $m_{i}(\textbf{z}) = \mathbb {E}[f_{i}(\textbf{z})]$ and $k_{i}(\textbf{z},\textbf{z}') = \mathbb {E}[(f_{i}(\textbf{z})-m_{i}(\textbf{z}))(f_{i}(\textbf{z}')-m_{i}(\textbf{z}'))]$ are the mean and the covariance function (kernel) of the process $f_{i}$, respectively. We follow a standard practice and assume that $m_{i}(\textbf{z})=0$; this implies that the latent process $f_{i}(\textbf{z})$ is fully specified by its kernel function.

For the collected covariates $\textbf{Z}=[\textbf{z}^{(1)},\textbf{z}^{(2)},\dots ,\textbf{z}^{(M)}]^{T}$ the GP prior over $\textbf{f}_{i}=[ f_{i}(\textbf{z}^{(1)}),f_{i}(\textbf{z}^{(2)}),\dots ,f_{i}(\textbf{z}^{(M)})]^{T}$, the vector of stacked latent function values, is given by $p(\textbf{f}_{i}\vert \textbf{z},\varvec{\phi }_{i}) = \mathcal {N}(\textbf{f}_{i}\vert \textbf{0},\textbf{K}_{i}),$ where $\varvec{\phi }_{i}$ denotes the GP kernel hyperparameters and $\textbf{K}_{i} = k_{i}(\textbf{Z},\textbf{Z})$. The likelihood can be expressed as $p(\varvec{\Theta }_{i}\vert \textbf{f}_{i})=\mathcal {N}(\varvec{\Theta }_{i}\vert \textbf{f}_{i},\sigma ^{2}_{i}\mathbb {I})$, where $\varvec{\Theta }_{i}=[\theta _{i}^{(1)},\theta _{i}^{(2)},\dots ,\theta _{i}^{(M)}]^{T}$ and $\mathbb {I}$ is the identity matrix. Marginalising over the latent variables gives the formula for the marginal likelihood

$$\begin{aligned} p(\varvec{\Theta }_{i})=\mathcal {N}(\varvec{\Theta }_{i}\vert \textbf{0},\textbf{K}_{i}+\sigma ^{2}_{i}\mathbb {I}). \end{aligned}$$

Under the Gaussian observation model the conditional posterior distribution of the latent variables is also Gaussian and is given by

$$\begin{aligned} p(\textbf{f}_{i}\vert \varvec{\Theta }_{i},\textbf{Z},\varvec{\phi }_{i})=\mathcal {N}(\textbf{K}_{i}(\textbf{K}_{i}+\sigma ^{2}_{i}\mathbb {I})^{-1}\varvec{\Theta }_{i}, \textbf{K}_{i} - \textbf{K}_{i}(\textbf{K}_{i}+\sigma ^{2}_{i}\mathbb {I})^{-1}\textbf{K}_{i} ). \end{aligned}$$

***Kernel specification***

In our experiments in Sect. 4.3.1 we will adopt the squared exponential (SE) kernel, which is one of the most popular kernels in the literature, mostly because it results in a smooth prior on the latent function. We will also allow for automatic relevance determination (ARD) (Neal 2012; Rasmussen and Williams 2006), which provides a built-in method of variable selection. ARD kernels have a separate length scale per predictor; the ARD SE kernel has the following form

$$\begin{aligned} k(\textbf{z},\textbf{z}') = \sigma ^{2}_{se} \exp \left( -\sum _{g=1}^{G}\frac{(z_{g}-z_{g}')^{2}}{2l_{g}^2}\right) , \end{aligned}$$

where the vector of kernel hyperparameters to be estimated is given by $\varvec{\phi }=( \sigma ^{2}_{se}, l_{1},\dots ,l_{G})^{T}$. The inverse of the length scale parameters $l_{g}$, $g=1,\dots ,G$, can be seen as the weight of the corresponding explanatory variable $z_{g}$, determining how relevant it is.

### Modified semi-automatic GP-ABC

GPs are designed for interpolation and do not extrapolate well (Brynjarsdóttir and O’Hagan 2014). ABC-SMC algorithms (semi-automatic or not) perturb parameter samples, meaning that values could be sampled from outside the GP training region, requiring the GPs to extrapolate. This can result in inference problems, e.g. inferring false modes of the ABC-posterior distribution (which we report in Sect. 4.3.1). To overcome the extrapolation issues we investigate two variations of the basic GP setting and assess what influence they have on ABC inference.

#### GP-ABC with convex hulls

Our first approach is to restrict the sampling region for the GP-ABC algorithm to the convex hull of the GP training region. This ensures that the samples remain within the training region. Specifically, we first create a convex hull over the GP training set. Then, when perturbing the parameter samples during ABC-SMC, we reject and resample those draws that fall outside of the training region. We note, however, that restricting the sampling region of the GP-ABC algorithm to the GP training region could introduce a bias to the ABC results. The resulting algorithm is the same as Algorithm 1, except for potential rejection-resampling done in Step 5.

#### Residual approach

An alternative approach to alleviating the extrapolation problem is based on combining GP regression with MVLR in a two-stage regression. A similar approach is proposed by Conti et al. (2009), who integrate the regression parameters out.

We first fit MVLR (23) as in the linear regression semi-automatic ABC from Sect. 3.3.1. Then, we train a zero-mean GP regression model on the residuals from the first-stage regression as follows

25 $$\begin{aligned} \hat{\epsilon }_{i} = f_{i}(\textbf{z}) + \xi _{i},\quad \xi _{i} {\mathop {\sim }\limits ^{iid}} \mathcal {N}(\cdot \vert 0,\omega _{i}^{2}), \end{aligned}$$

where $\hat{\epsilon }_{i}=\theta _{i}-\hat{\theta }_{i}=\theta _{i}- (\hat{\beta }_0^{(i)}+{\varvec{\hat{\beta }}}^{(i)}\textbf{z})$ is the residual from (23), while $f_{i}$ follows a GP (see Sect. 3.3.2).

## ABC comparison results on the toy problem

In this section we compare the performance of the ABC algorithms discussed in Sect. 3 on the simple drift-diffusion SDE from Sect. 2.5. The aim of this analysis is to select the most appropriate ABC algorithm for inference in the cell movement model proposed in Sects. 2.1 and 2.2. As already emphasised, an exhaustive comparative evaluation of the ABC methods directly on the complex cell movement model is practically infeasible due to the high computational costs.

### Simulation setting

We generate synthetic data by solving numerically (14) by the Euler-Maruyama method as discussed in Sect. 2.5.1 with the model parameters set to $D=2$ and $\alpha =1$. Hence, the observed data ${\varvec{y}}=\{{\varvec{x}}^{(j)}\}_{j=1}^{N_{S}}$ consist of $N_{S}=100$ trajectories generated from (14).

Because Devlin et al. (2019) demonstrated that the inference accuracy for a MVLR model depends crucially on the value of *T*, we want to investigate whether the performance of the ABC schemes reveals a similar dependence for our SDE model 14. Therefore, we consider three values of *T*: $T=0.05$, 5 and 500.

### Implementation of ABC algorithms

Each ABC algorithm is run with $N_a=1000$ parameter acceptances. The semi-automatic approaches from Sects. 3.3 and 3.4 utilise pilot runs with calibration sets of $M=1000$ draws. That training set comes from the last intermediary distribution of a pilot run of the ABC-SMC with adaptive weights of Prangle (2017) with three iterations.

For the rejection ABC and ABC-SMC algorithms we use the $l^{2}$ norm, while for the semi-automatic algorithms we adopt the $l^{1}$ norm as the distance function. The reason for using different norms is somewhat arbitrary and motivated by different lengths of summary statistics vectors. In the former case we compare MSD vectors of length 100, while in the latter case there are only two second-stage summary statistics corresponding to posterior means for the two parameters. Below we report configurations for specific algorithms.

#### Rejection algorithm

We set higher values of the tolerance parameter $\varepsilon$ for larger *T* values, which is motivated by the properties of the MSD, see Sect. 2.6.2. Moreover, we investigate how the ABC-posterior distributions change for a schedule of decreasing tolerance values reported in Table 2.

**Table 2:** Rejection ABC: tolerance values for different values of *T*

T	$\varepsilon$ values			
0.05	2	1	0.5	0.25
5	200	100	50	25
500	200000	100000	50000	25000

#### ABC-SMC

We consider the ABC-SMC algorithm of Beaumont (2010) with the deterministic tolerance schedules reported in Table 3. As for the rejection algorithms, we condition the tolerance values on the value of *T*. We initialise with a fairly large value as is typically done for ABC-SMC algorithms.

**Table 3:** ABC-SMC: tolerance schedules for different values of *T*

T	$\varepsilon _{1}$	$\varepsilon _{2}$	$\varepsilon _{3}$	$\varepsilon _{4}$	$\varepsilon _{5}$	$\varepsilon _{6}$
0.05	8	4	2	1	0.5	0.25
5	800	400	200	100	50	25
500	800000	400000	200000	100000	50000	25000

#### ABC-SMC with adaptive distance function

We run the algorithm with eight iterations. In the first iteration we set the tolerance value large enough to accept all parameter samples. In subsequent iterations we use the $\tau$th quantile of the previous accepted distance values as tolerance values. For the examples tested by Prangle (2017) the quantile value $\tau =0.5$ performed best. In our experimentations, however, such a small value led to too low acceptance rates causing the algorithm to be very slow (most likely due to using the MSD as our summary statistics). Therefore, we take the smallest value of $\tau$ that allows for reasonable run time, for each of the three different values of *T*: for $T=0.05$, 5 and 500, we set $\tau =0.9$, 0.5 and 0.6, respectively.

#### Semi-automatic ABC

In the main run of the semi-automatic approach we adopt the ABC-SMC with adaptive weights algorithm of Prangle (2017). Hence, as before, we weight each summary statistics by an estimate of its MAD. We also take the same quantile values $\tau$ (in the first iteration we set the tolerance at the $\tau$th quantile of the draws from the last iteration of the pilot run – the one used to fit the regression model).

#### Residual approach

We initialise the residual approach in the same way we initialise semi-automatic ABC. Having fitted MVLR, we retrieve the residuals of the parameter values, to which we then fit a GP regression model. The second-stage summary statistics are then calculated as a sum of the fitted values from the MVLR and the GP regression model. Note that for the semi-automatic and the residual approach we found good acceptance rates could be obtained by setting $\tau =0.5$.

### Results

#### Exact posterior parameter distribution

We start our analysis by discussing the results for the gold-standard, the exact posterior distribution, which we plot on a uniform mesh of 1000$\times$1000 points over the prior domain. At each of the mesh points we calculate the population likelihood (17) for the $N_S$ trajectories, the shape of which matches that of the population posterior due to uniform priors for *D* and $\alpha$.

**Fig. 5:**
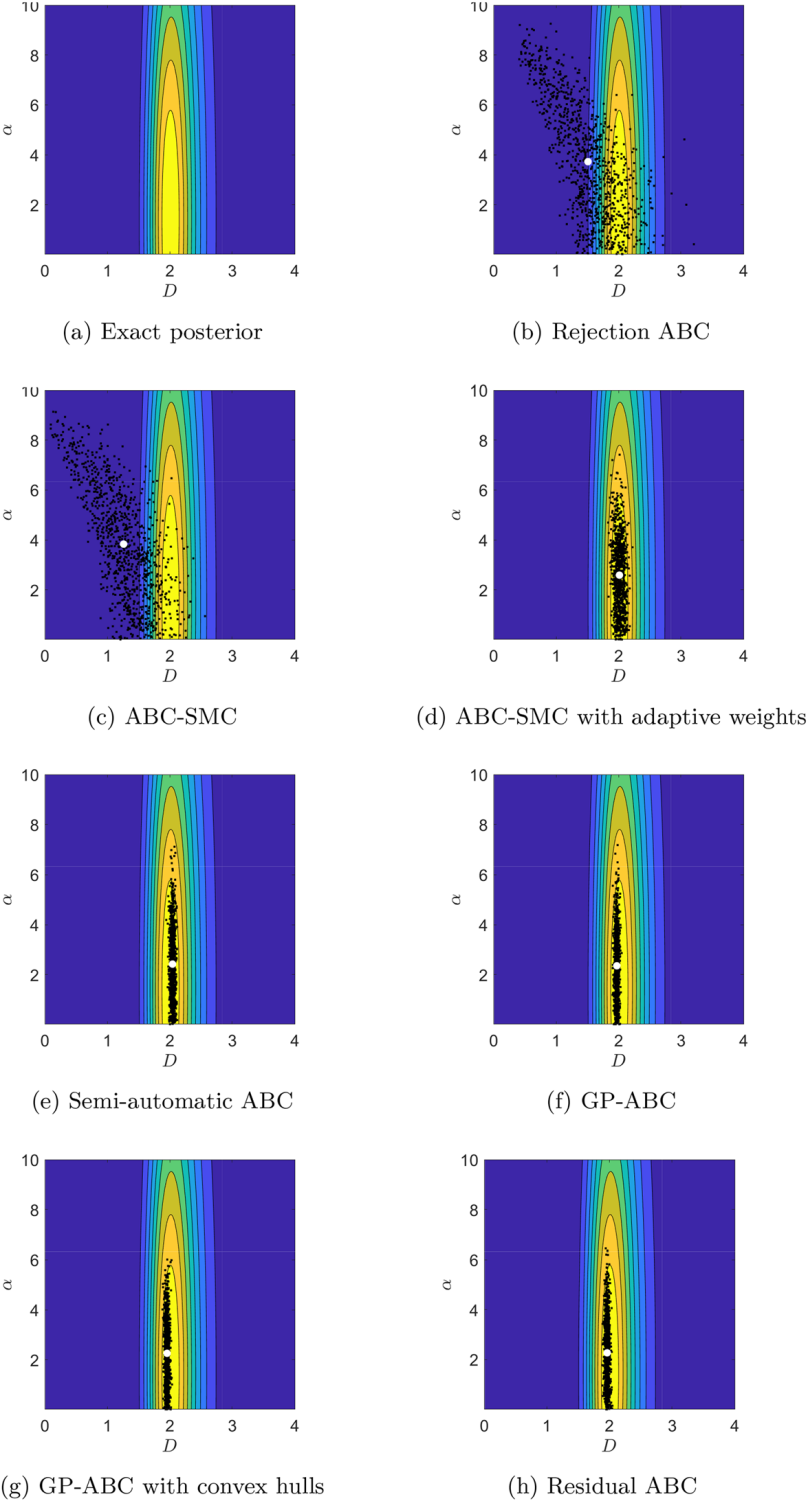
Contour plots of the exact posterior distribution, superimposed with draws from the ABC algorithms at *T*=0.05 s. White dot: ABC posterior mean

**Fig. 6:**
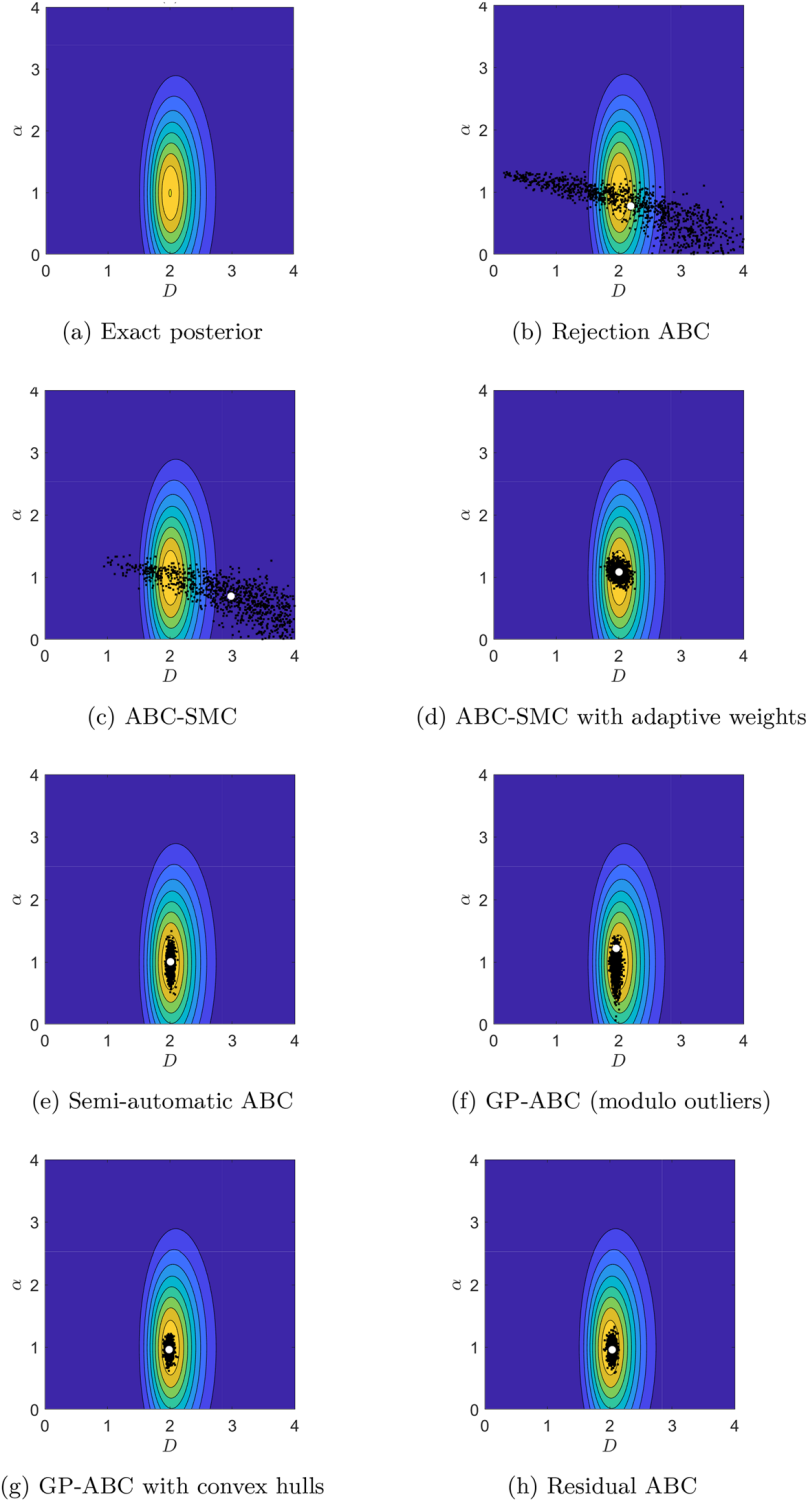
Contour plots of the exact posterior distribution, superimposed with draws from the ABC algorithms at *T*=5 s. White dot: ABC posterior mean

**Fig. 7:**
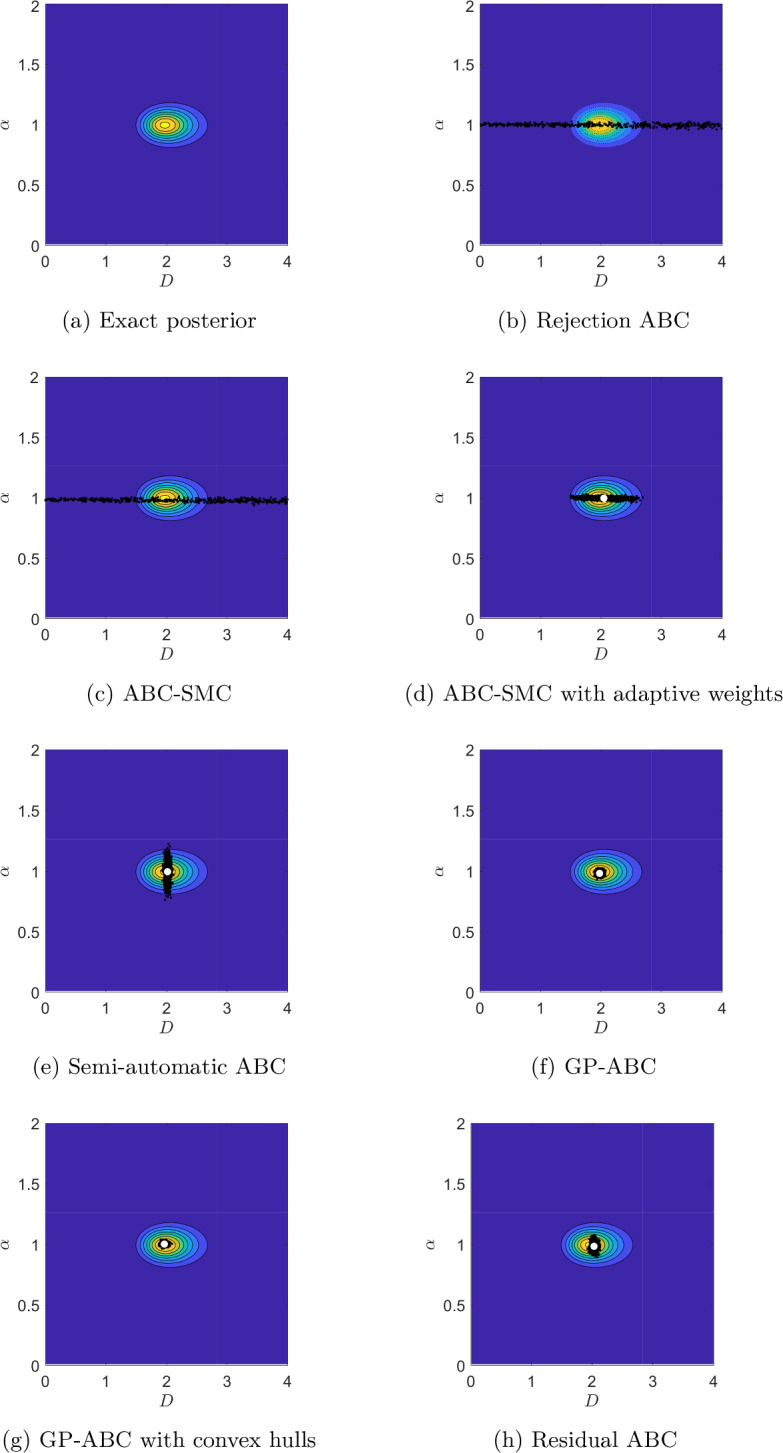
Contour plots of the exact posterior distribution, superimposed with draws from the ABC algorithms at *T*=500 s. White dot: ABC posterior mean

Figures 5a, 6a and 7a present contour plots of the likelihood for the values $T=0.05$, 5 and 500, respectively. As expected, the shape of the likelihood heavily depends on *T*. Similar to Devlin et al. (2019) for a small value of *T*, $\alpha$ is difficult to infer accurately, with the likelihood being very dispersed in $\alpha$. As we increase *T*, the likelihood becomes more isometric and concentrated around the true parameter values. However, increasing *T* indefinitely will not improve inference as too high a *T* value will lead to identification problems for *D* as the MSD will be dominated by the drift term, as we demonstrate in Appendix C.1. The chosen large value for *T* of 500 is still moderate in this regard so *D* is practically identifiable in this case.

**Fig. 8:**
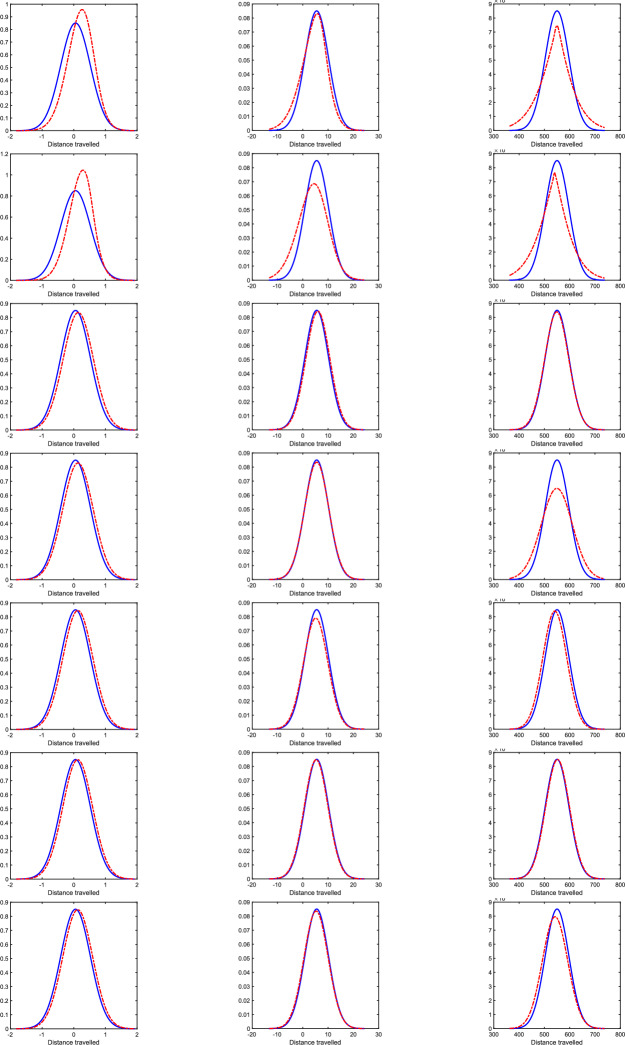
True (blue solid lines) and predictive distributions (red dashed lines) at $T=0.055$s (left column), $T=5.5$s (centre column) and $T=550$s (right column).The different rows correspond to the different ABC methods, from top to bottom: Rejection ABC, ABC-SMC, ABC-SMC with adaptive weights, Semi-automatic ABC, GP-ABC, GP-ABC with convex hulls and Residual ABC

We focus on comparing the results from the ABC algorithms with the exact posterior visually, by superimposing the draws from each algorithm with the contour plots of the exact posterior in Figs. 5, 6 and 7 for $T=0.05$, 5 and 500, respectively. For all the values of *T* the sampling domain is $[0,10]^{2}$, however, for the middle and large value of *T* the posterior distributions become very concentrated, so we “zoom in” and present the results on $[0,4]^{2}$. Table 4 quantitatively summarises the results in terms of percentage relative errors of means and variances from ABC with respect to the corresponding values for the exact posterior. To provide ballpark figures of the uncertainty associated with the presented error estimates we report their approximate standard deviation based on Gaussian error propagation. We assume no correlation between the moments of the ABC posteriors and those of the exact posterior (this assumption can only lead to slightly overestimated uncertainty of the presented errors).

**Table 4:** Relative percentage errors between the ABC posterior means and variances and those for the exact posterior for *D* and $\alpha$, together with the corresponding standard errors (SE)

Method	Mean *D*		Variance *D*		Mean $\alpha$		Variance $\alpha$	
	Estimate	SE	Estimate	SE	Estimate	SE	Estimate	SE
*T*=0.05 s								
Rejection	−27.92%	2.64%	23454.60%	308.50%	−19.93%	5.56%	7097.26%	170.53%
SMC	−39.85%	2.69%	20242.61%	286.69%	**−17.79%**	5.33%	6691.40%	165.65%
SMC adj	**−4.05%**	0.34%	328.27%	41.60%	−44.41%	4.14%	2673.38%	105.86%
Semi-auto	−4.53%	0.20%	−14.35%	18.60%	−30.88%	4.22%	3458.04%	119.90%
GP	−5.00%	0.19%	−25.05%	17.40%	−41.61%	3.94%	**2524.57%**	102.98%
GP conv	−5.00%	0.19%	−35.76%	16.11%	−43.33%	4.06%	2605.74%	104.56%
Residual	−5.00%	0.20%	**−3.64%**	19.73%	−41.40%	4.10%	2754.55%	107.39%
*T*=5 s								
Rejection	4.48%	4.18%	85163.16%	586.94%	−36.57%	3.42%	1900.36%	89.90%
SMC	41.52%	3.15%	65163.16%	513.51%	−43.08%	3.27%	1536.66%	81.32%
SMC adj	−4.54%	0.43%	636.84%	54.56%	−12.17%	1.04%	81.85%	27.11%
Semi-auto	−3.12%	0.20%	**−15.79%**	18.45%	−29.25%	1.75%	445.55%	46.95%
GP	−3.12%	0.19%	−26.32%	17.25%	**−4.85%**	6.43%	10629.22%	208.21%
GP conv	**−2.65%**	0.19%	−26.32%	17.25%	−12.99%	0.96%	**45.48%**	24.24%
Residual	**−2.65%**	0.20%	**−15.79%**	18.45%	−15.43%	0.61%	−83.63%	8.13%
*T*=500 s								
Rejection	125.83%	8.92%	835029.31%	1836.92%	−0.65%	0.20%	261.45%	38.22%
SMC	114.75%	8.60%	738046.55%	1726.97%	−2.66%	0.17%	140.96%	31.20%
SMC adj	−1.29%	1.29%	7443.10%	174.58%	**0.35%**	0.14%	**20.48%**	22.06%
Semi-auto	**1.12%**	0.21%	**7.76%**	20.87%	8.38%	0.92%	10743.37%	209.31%
GP	**1.12%**	0.19%	−24.57%	17.46%	**0.35%**	0.10%	−90.36%	6.24%
GP conv	**1.12%**	0.20%	−13.79%	18.66%	**0.35%**	0.10%	−89.16%	6.62%
Residual	**1.12%**	0.20%	−13.79%	18.66%	**0.35%**	0.14%	**20.48%**	22.06%

Compared methods are: rejection ABC (Rejection), ABC-SMC (SMC), ABC-SMC with adaptive weights (SMC adj), semi-automatic ABC with MVLR (Semi-auto), GP-ABC (GP), GP-ABC with convex hulls (GP conv), residual ABC (Residual). Negative numbers mean that the ABC value is smaller than the corresponding exact posterior value. Lowest estimate values (for each quantity, for each value of *T*) in bold

**Table 5:** Percentage coverage of $95\%$ highest posterior density regions *C* and the mean of the widths of the 95% highest density intervals (HDI) for *D* and $\alpha$

Method	$T=0.05$			$T=5$			$T=500$		
	$\langle \vert HDI_{D}\vert \rangle$	$\langle \vert HDI_{\alpha }\vert \rangle$	*C*	$\langle \vert HDI_{D}\vert \rangle$	$\langle \vert HDI_{\alpha }\vert \rangle$	*C*	$\langle \vert HDI_{D}\vert \rangle$	$\langle \vert HDI_{\alpha }\vert \rangle$	*C*
Semi-auto	0.88	6.22	99	0.11	0.58	84	0.11	0.30	90
GP	0.11	4.17	90	0.16	4.94	98	0.16	0.21	96
GP conv	0.10	4.21	93	0.14	0.37	88	0.14	0.05	97
Residual	0.18	4.71	94	0.15	0.40	90	0.14	0.13	100

Compared methods are: Semi-automatic ABC with MVLR (Semi-auto), GP-ABC (GP), GP-ABC with convex hulls (GP conv), and residual ABC (Residual). For $T=5$ the increased width of the credible interval for GP is caused by the bimodality of the corresponding ABC posterior distribution; see main text, Sect. 4.3.3

##### Rejection ABC

Figures 5b, 6b and 7b present the draws from the rejection ABC algorithm with the lowest tolerance values from Table 2 for $T=0.05$, 5 and 500, respectively. Figures 14, 15 and 16 in Appendix C illustrate the results for all the considered tolerance values from Table 2. Not surprisingly, rejection ABC leads to poor approximations of the exact posterior distributions, as confirmed by the extremely large errors reported in Table 4. This might be due to using the MSD as the summary statistics, which might not be informative enough.

##### ABC-SMC

Samples from the final intermediary distributions of the ABC-SMC algorithm for $T=0.05$, 5 and 500 are shown in Figs. 5c, 6c and 7c, respectively. We refer the reader to Figs. 17, 18 and 19 in Appendix C for an overview of the convergence over the sequence of intermediary distributions with the tolerance schemes from Table 3. The final ABC posterior distributions are very similar to those from the rejection algorithm – as we would expect, given the same $\varepsilon$ values used in both cases. There is still a noticeable discrepancy between the ABC and true posterior distributions, resulting in substantial errors (see Table 4).

##### ABC-SMC with adaptive distance function

Figures 5d, 6d and 7d show the draws from the ABC-SMC algorithm with adaptive weighting (Prangle 2017) for $T=0.05$, 5 and 500, respectively. We find that scaling each MSD value by an estimate of its MAD generally improves inference compared to the standard SMC approach, resulting in much lower errors (see Table 4). For the small value of *T*, the correlation between $\alpha$ and *D* is better captured, while for the large value of *T* we are able to practically identify $\alpha$ and *D*, as demonstrated by negligible errors for the posterior means of both parameters. For the middle value of *T*, both $\alpha$ and *D* are inferred relatively accurately. However, for all the values of *T* the algorithm struggles to capture the true parameter uncertainty, with the ABC-posterior variances being much larger than the true ones (see Table 4).

##### Semi-automatic ABC with MVLR

Figures 5e, 6e, 7e illustrate the samples from semi-automatic ABC for $T=0.05$, 5 and 500, respectively. We see that using the second-stage summary statistics (estimates of the posterior means) improves the inference over all the previous approaches based on the first-stage summary statistics (MSD), especially for *D*. Still, it remains problematic to accurately estimate parameter uncertainty, with the ABC-posterior variances generally being either too disperse (for $\alpha$) or too tight (for *D*). For the latter, however, the standard errors are larger than the absolute values of the corresponding estimates, signalling that the apparent underdispersion of the ABC posteriors is not significant. Compared with ABC-SMC with adaptive distance function, the variance of the semi-automatic ABC posteriors is higher for $\alpha$ but lower for *D* (see Table 4). This may be expected as the MSD is linear in *D* while quadratic in $\alpha$, and so we would expect using linear regression to extract more reliable values for *D* than for $\alpha$.

##### GP-ABC

The samples from GP-ABC for $T=0.05$, 5 and 500 are given in Figs. 5f, 6f and 7f, respectively. For the middle value of *T* there was a small group of outlier draws concentrated around $D=2, \, \alpha =7$, which is not illustrated in Fig. 6f for the scale consistence with other figures. This explains the inflated error for the ABC-posterior variance of $\alpha$ for $T=5$. For the remaining values of *T* the difference with respect to the MVLR semi-automatic algorithm is moderate, with the GP-ABC algorithm tending to underestimate the posterior variance of *D* a bit more often (see Table 4). We note, however, that the standard errors of the variance estimates for *D* are substantial, meaning that the recorded underdispersion is not significant. For the middle and large values of *T*, the location of the GP-ABC posterior for $\alpha$ is better than for the MVLR semi-automatic ABC approach.

##### GPs with convex hulls

Figures 5g, 6g and 7g present the samples obtained with GP-ABC with convex hulls for $T=0.05 \, \textrm{s}$, 5 and 500, respectively. We find that restricting the sampled parameter values using convex hulls removes the outliers present in the GP-ABC posterior distributions observed for $T=5$. This confirms our conjecture that the problem with practical identification was caused by unsatisfactory extrapolation from the GP training region. However, the ABC-posteriors are still underdispersed in *D* and, for the large *T*, also in $\alpha$ compared to the exact posterior (see Table 4). However, again, the underestimated variance for *D* is subject to considerable standard errors – for the large *T* even greater then the absolute value of the corresponding estimate – suggesting that there is substantial simulation noise affecting the recorded results.

##### The residual approach

The samples from the residual ABC algorithm for $T=0.05 \, \textrm{s}$, 5 and 500 are given in Figs. 5h, 6h and 7h, respectively. The ABC-posterior distributions for the small and middle value of *T* seem rather similar to those from GP-ABC with convex hulls, while for the large value of *T* the ABC-posterior is more dispersed in $\alpha$. The uncertainty in *D* remains underestimated for the middle and large *T*, but is captured relatively well for the small value of *T* (see Table 4). This suggests that the potential bias induced by introducing the convex hull truncation might be negligible in our case. As for semi-automatic ABC with MVLR the standard errors for the variances for *D* are larger than the absolute values of the corresponding estimates, which signals that the underestimation of the variance is likely due to the simulation noise.

#### Predictive distributions

Next, we inspect the predictive posterior distributions in output space. The out-of-sample posterior distribution of *x* for a future timepoint $t^*$, $x[t^*]$, is given by

26 $$\begin{aligned} p(x[t^*]\vert {\varvec{y}})= & \int p(x[t^*]\vert \alpha ,D)p(\alpha ,D\vert {\varvec{y}}) \end{aligned}$$

where ${\varvec{y}}$ is the set of data used for training. For a conjugate prior the posterior distribution

27 $$\begin{aligned} p(\alpha ,D\vert {\varvec{y}}) \;= \; \frac{L({\varvec{y}}\vert \alpha ,D) p(\alpha ,D)}{ \int L({\varvec{y}}\vert \alpha ,D) p(\alpha ,D) d\alpha dD } \end{aligned}$$

and the integral in equation (26) can be worked out analytically. However, for a uniform prior, it is easier to compute the integral in equation (26) numerically with the Monte Carlo method:

28 $$\begin{aligned} p(x[t^*]\vert {\varvec{y}})= & \frac{ p(x[t^*]\vert \alpha _i,D_j)L({\varvec{y}}\vert \alpha _i,D_j) }{ \sum _{i,j} p(x[t^*]\vert \alpha _i,D_j)L({\varvec{y}}\vert \alpha _i,D_j) } \end{aligned}$$

where the summation $\sum _{i,j}$ extends over a dense mesh in parameter space and the likelihood is obtained from equation (17). When the likelihood is unknown (or assumed unknown, for method evaluation), the predictive distribution is approximated by

29 $$\begin{aligned} p(x[t^*]\vert {\varvec{y}})\approx & \tilde{p}(x[t^*]\vert {\varvec{y}}) \; =\; \frac{1}{M} \sum _{i=1}^M p(x[t^*]\vert \alpha _i,D_i) \end{aligned}$$

where $\{\alpha _i,D_i\}_{i=1}^M$ is our approximate sample from the unknown posterior distribution $p(\alpha ,D\vert {\varvec{y}})$, obtained with the various ABC samplers discussed earlier. Inserting the expression from equation (15), we get:

30 $$\begin{aligned} p(x[t^*]\vert {\varvec{y}})\approx & \frac{1}{M} \sum _{i=1}^M \frac{1}{\sqrt{4\pi D_i t^*}}\exp \left( \frac{- (x[t^*]-\alpha _i t^*)^2}{4D_i t^*}\right) . \end{aligned}$$

We have repeated the training simulations three times, for different time intervals [0, *T*] with $T\in \{0.05,5,500\}$, and made out-of-sample predictions $10\%$ above that time interval, that is, we set $t^* = 1.1T$. The results are shown in Fig. 8.

A comparison of Figs. 5, 6, 7 and 8 shows very good agreement. Rejection ABC and ABC-SMC, for which the ABC posterior samples are not well aligned with the parameter domain of high likelihood (Panels b-c in Figs. 5, 6 and 7) also show the largest deviation between the estimated and true predictive distributions (top two rows in Fig. 8). The other ABC algorithms, whose posterior parameter samples are much better aligned with the regions of high likelihood, also achieve a much better agreement between the estimated and true predictive distributions. Of those the poorest agreement has been found for the semi-automatic ABC algorithm at the largest value of *T* (Fig. 8, 4th row, right column). This tallies with Fig. 7e, which shows several sampled $\alpha$ values falling into the tails of the high likelihood region.

#### Coverage of HDI intervals

We have also estimated the coverage of the highest posterior density intervals (HDI) for the best performing methods. To this end we have repeated the ABC simulations $K=100$ times. For each simulation we have estimated the 95% highest density interval using the package bayestestR (Makowski et al. 2019). We have then established how often the true parameters were included in the central 95% interval. The coverage is given by the percentage of simulations that included the true parameters in this central 95% posterior interval. For a reliable sampler, the coverage should be close to 95%. We also recorded the width of the central 95% posterior interval; this gives an indication of how compact the 95% posterior support is. The results can be found in Table 5. We note that in a few cases where the posterior distribution is bimodal, this measure is misleading as it does not allow for the low posterior probability region between the modes; see e.g. Figure 2.2 in Gelman et al. (2014) for an illustration. This explains some of the outliers in Table 5. However, the coverage is, in general, close to the target value of $95\%$, suggesting that our selected ABC samplers achieve a reliable approximation of the true posterior distribution.

#### Practical identification and accuracy measures

In Sect. 4.3.1 we have analysed the performance of ABC algorithms compared with the gold-standard exact posterior distribution. However, as we have seen in Sect. 4.3.1, even for the exact posterior whether both parameters are practically identifiable depends on the value of *T*. For ABC these issues with practical identification can be further amplified by choosing the MSD as summary statistics. Moreover, we have observed that substantial simulation noise can make capturing the true uncertainty of both parameters challenging, even for more advanced ABC schemes (ABC-SMC with adaptive weights and semi-automatic ABC algorithms). This raises different questions to that of comparing ABC with the exact posterior. How good a job do ABC algorithms do in practically identifying the parameters? And, how to measure practical identification accuracy in the first place? To address these questions, in Appendix D we introduce two accuracy measures, which we then use to compare ABC algorithms in terms of their practical identification performance.

## Self-generated gradient model simulation study

This section presents our main results on inferring key parameters of the proposed cell movement model (see Sect. 2) using ABC. As we discuss previously, we focus on two parameters of the model, *D* and $\nu$, as they are physiologically most relevant.

### ABC implementation

To illustrate the performance of the ABC methods we will focus initially on the residual ABC algorithm (see Sect. 3.4.2), as it was found to be one of the best approaches among those tested in Sect. 4. We set the nominal values for *D* and $\nu$ to $3 \, \mu \mathrm{m^2/s}$ and $31.57 \, \mu \mathrm{m/s}$, respectively, and adopt a triangular distribution^2^ as the prior on *D* and $\nu$, with maximum and minimum values equal to double and half the nominal values, respectively. For the vector of summary statistics used during the initial run of ABC-SMC with adaptive distance function, we use the ensemble MSD calculated at the $N-1$ non-zero time points. The observed vector of summary statistics will be the ensemble MSD calculated using the nominal values from Table 1.

### ABC results

**Fig. 9:**
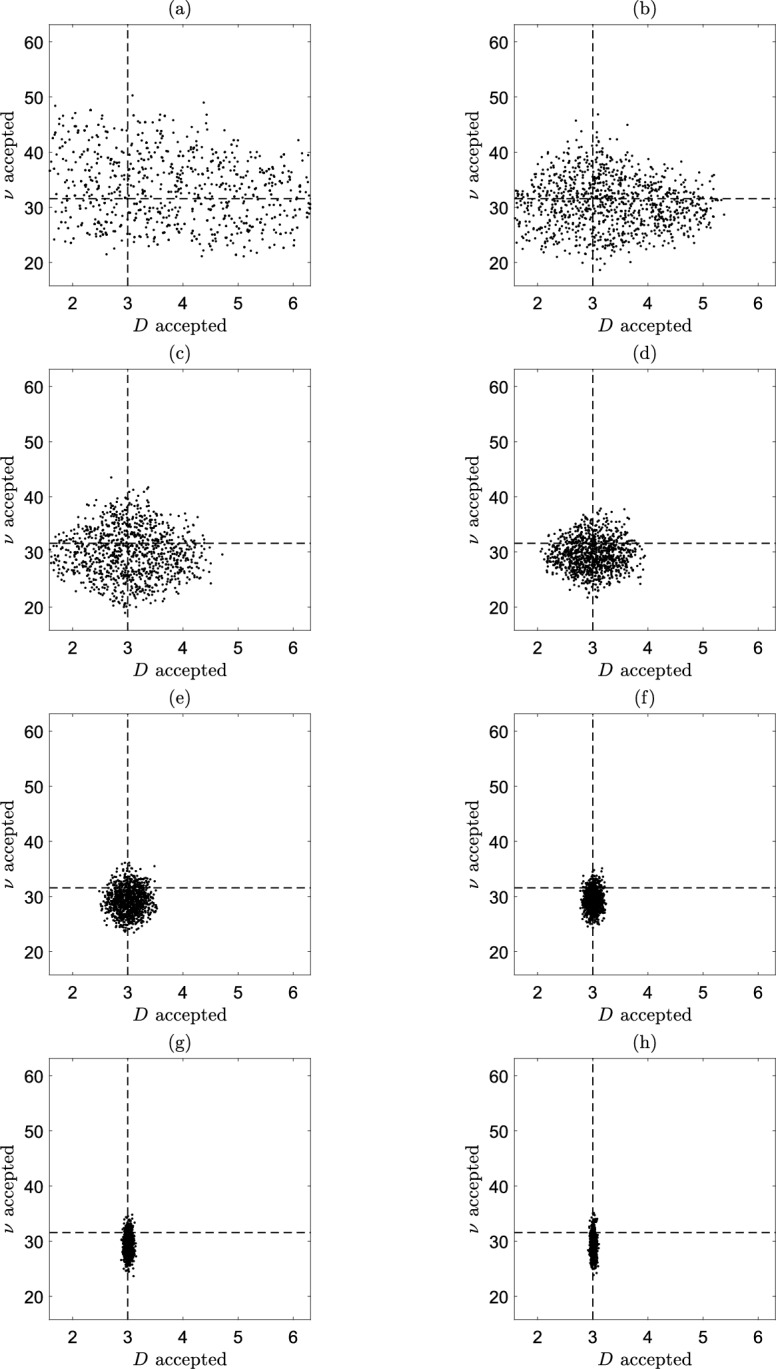
Samples from the joint posterior distributions for *D* and $\nu$ using the residual ABC algorithm. The value of $\varepsilon$ decreases as we progress through (**a**) to (**h**). The dashed lines correspond with the true values of *D* and $\nu$. These experiments were for $N_S=100$, $N=500$ and $T=19800 \, \textrm{s}$, with the parameter values given in Table 1

Figure 9 presents samples from the estimated joint posterior distribution for *D* and $\nu$ after 8 iterations of the residual ABC algorithm, where the dashed lines indicate the true values of *D* and $\nu$. Although we do not have access to the exact joint posterior distribution, we find that both *D* and $\nu$ are inferred relatively well from the final estimated joint posterior distribution.

**Fig. 10:**
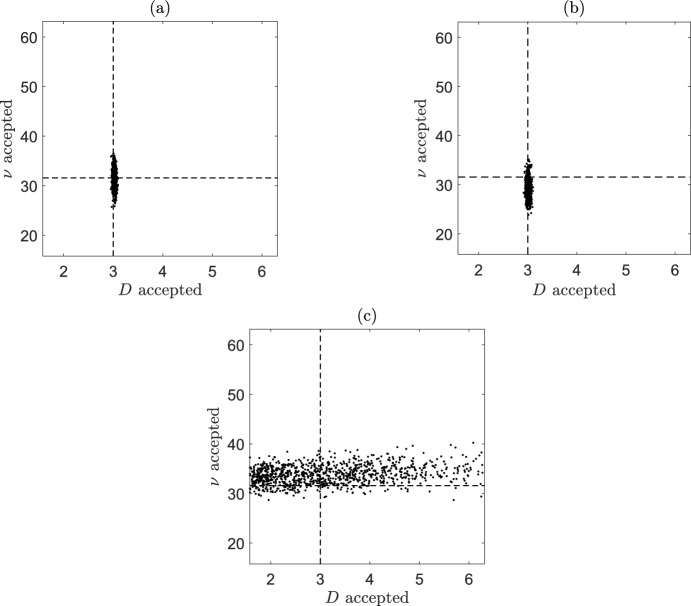
Samples from the joint posterior distributions for *D* and $\nu$ using the residual ABC algorithm, where the summary statistics is taken to be the first ten MSD points (**a**), all the MSD points (**b**), and the final ten MSD points (**c**). The dashed lines correspond with the true values of *D* and $\nu$. These experiments were for $N_S=100$, $N=500$ and $T=19800 \, \textrm{s}$, with the parameter values given in Table 1

The results from our toy problem simulation study in Sect. 4.3.1 showed that taking a smaller value of *T* generally improved the inference of the diffusion coefficient, while taking a larger value of *T* generally improves the inference of the drift velocity. Here, we test re-running the residual ABC algorithm where we calculate the ensemble MSD as before, but the summary statistics will be taken to be the first and final ten MSD points. If the MSD for the self-generated gradient problem is similar in form to that for the toy problem, then the behaviour of the MSD for short time intervals will be dominated by the random motility parameter *D*, but over longer time intervals the behaviour of the MSD is determined by the chemotaxis velocity parameter $\nu$. We would expect using the first ten MSD points to improve the inference of *D* and the final ten MSD points to improve the inference of $\nu$. Figure 10 shows a plot of the residual ABC results when we use the first ten MSD points, all the MSD points (note that this is the same plot as Fig. 9h), and the last ten MSD points as the summary statistics. When we use the final ten MSD points, *D* becomes unidentifiable as we would expect. For $\nu$, however, the marginal posterior distributions appear similar using the three different summary statistics and we do not find an improvement in the inference of $\nu$ when we use the final ten MSD points. This is likely due to the chemotactic term of our chemotaxis model being much more complex than the simple drift term considered in the toy model problem.

**Fig. 11:**
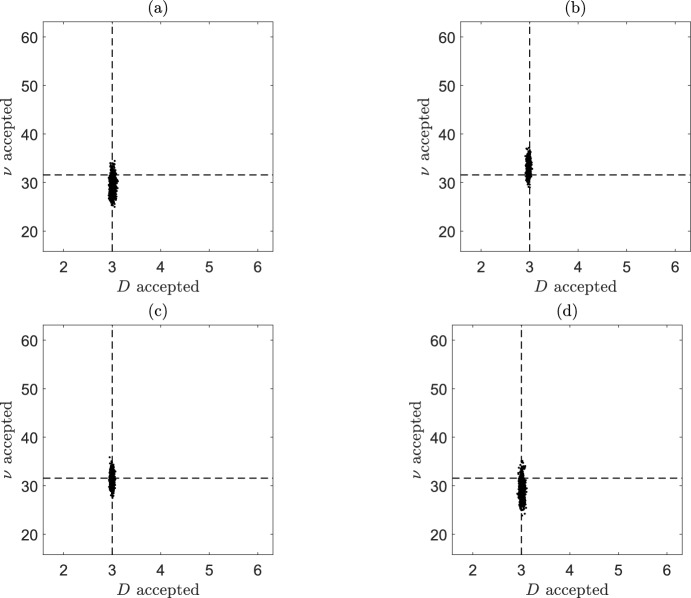
Samples from the joint posteriors using **a** Semi-automatic **b** GP **c** GP-Convex Hulls and **d** Residual ABC methods

**Fig. 14:**
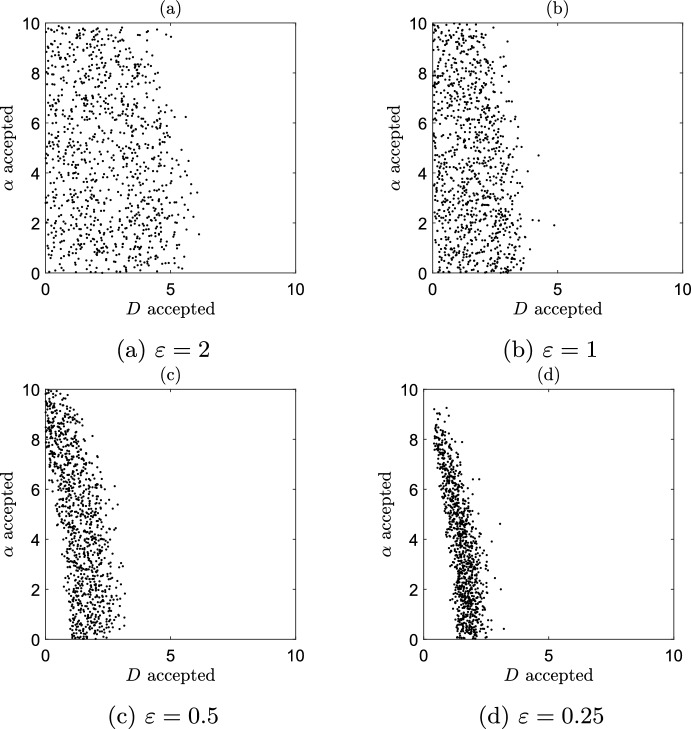
Rejection ABC: samples from the ABC-posterior distributions for $T=0.05 \, \textrm{s}$ and different $\varepsilon$ values

**Fig. 15:**
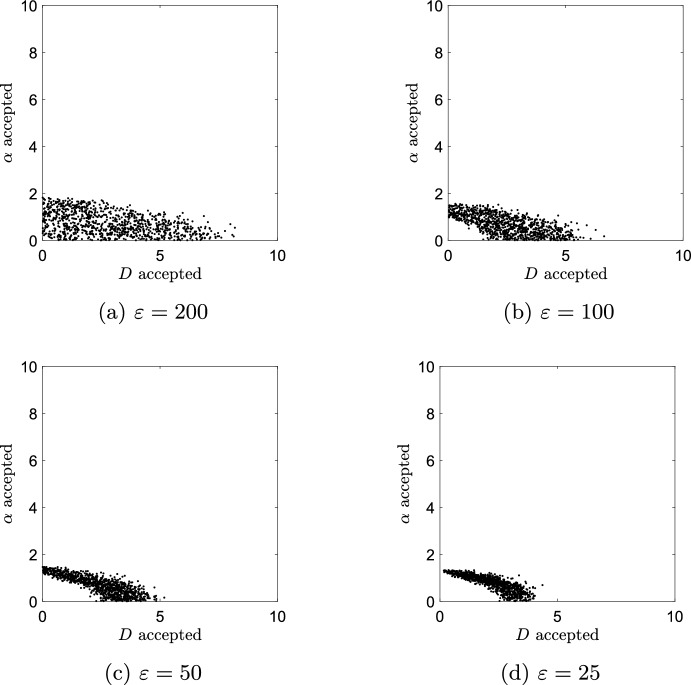
Rejection ABC: samples from the ABC-posterior distributions for $T=5 \, \textrm{s}$ and different $\varepsilon$ values

**Fig. 16:**
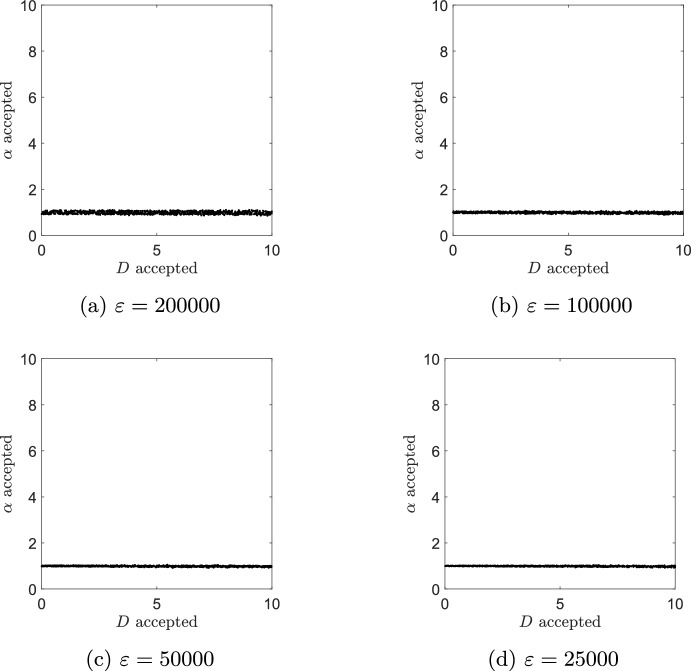
Rejection ABC: samples from the ABC-posterior distributions for $T=500 \, \textrm{s}$ and different $\varepsilon$ values

**Fig. 17:**
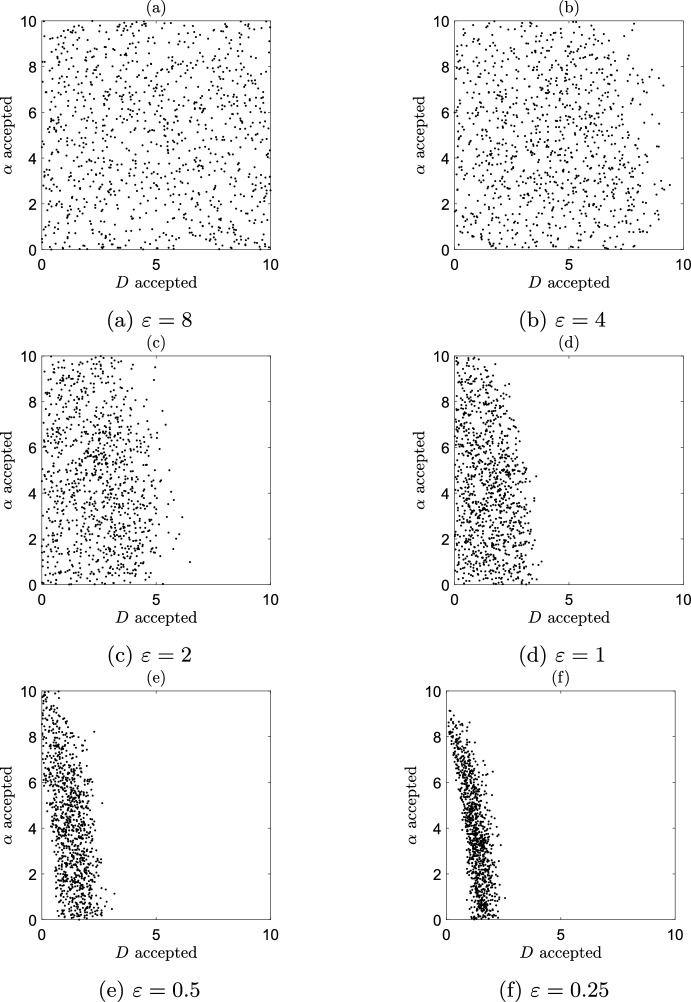
ABC-SMC: samples from subsequent intermediary joint distributions for $T=0.05 \, \textrm{s}$

**Fig. 18:**
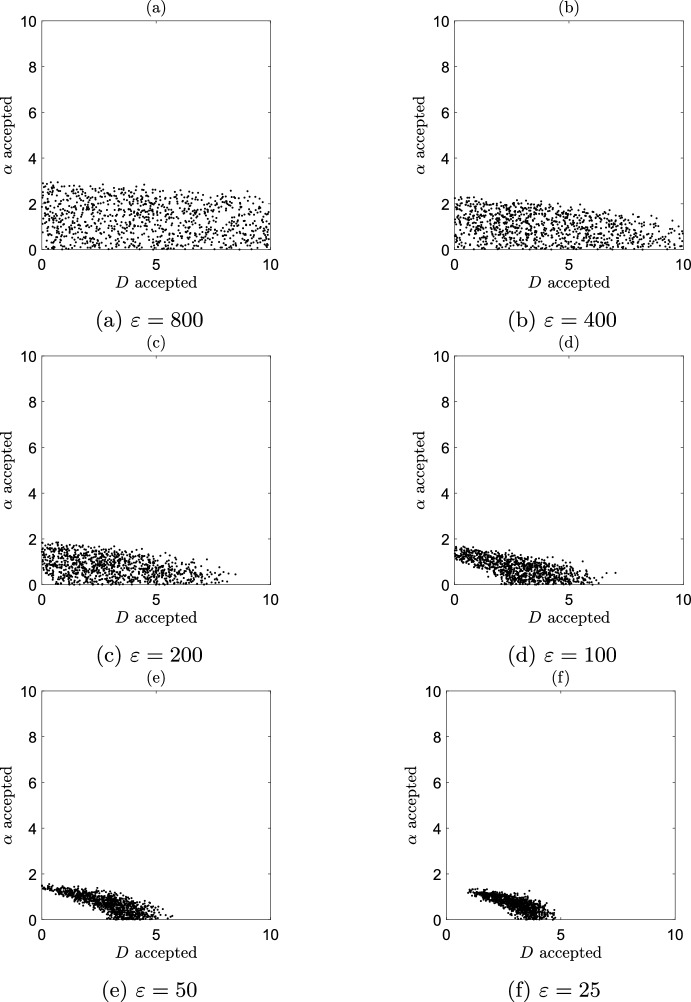
ABC-SMC: samples from subsequent intermediary joint distributions for $T=5 \, \textrm{s}$

**Fig. 19:**
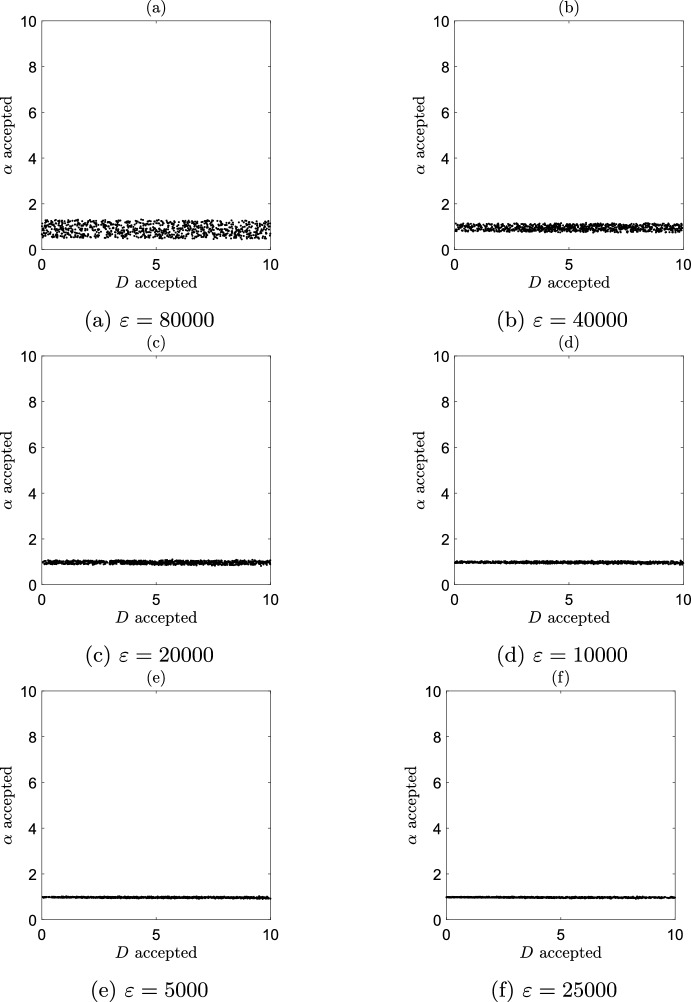
ABC-SMC: samples from subsequent intermediary joint distributions for $T=500 \, \textrm{s}$

In Table 6 and Fig. 11 the results using the residual ABC algorithm are compared to those obtained using the three other best performing methods identified in Sect. 4.3.1. In Table 6 we observe that the relative error in the posterior mean estimate for *D* is generally lower than that for the chemotaxis parameter $\nu$. This is likely to to due to the more complex nature of this term in the self-generated gradient model. In general, all four methods perform well in terms of accuracy and there is no evidence of any systematic bias in the estimates of the model parameters. While our focus has been on the relative accuracy of the considered ABC algorithms, our observations are that the four methods considered in this section produce their estimated posterior distributions over a similar time period.

**Table 6:** Percentage errors in ABC posterior means and relative MSE for self-generated gradient study

Method	*D*		$\nu$		
	Estimate	SE	Estimate	SE	MSE
Semi-auto	0.37%	$1.01\times 10^{-3}$ %	$-$6.79%	$5.51 \times 10^{-3}$ %	$8.22\times 10^{-3}$
GP	$-$0.67%	$6.59\times 10^{-4}$ %	5.72%	$3.82\times 10^{-3}$ %	$4.28\times 10^{-3}$
GP conv	$-$0.10%	$7.11\times 10^{-4}$ %	0.21%	$3.84\times 10^{-3}$ %	$1.53\times 10^{-3}$
Residual	0.36%	$9.64\times 10^{-4}$ %	$-$6.17%	$5.21\times 10^{-3}$ %	$7.66\times 10^{-3}$

Compared methods are: Semi-automatic ABC with MVLR (Semi-auto), GP-ABC (GP), GP-ABC with convex hulls (GP conv), and residual ABC (Residual)

## Discussion

In this paper we have first proposed a novel discrete-continuum biophysical model describing self-generated collective cell chemotaxis induced by the diffusion of a local chemoattractant. The cells move collectively according to the drift-diffusion SDE (1) and the evolution of the concentration of the chemoattractant is modelled using the PDE (9). Second, we have studied the inverse problem, inferring the two model parameters most critical for collective cell movement, *D* and $\nu$. Since the complexity of our biophysical model renders likelihood-based statistical inference infeasible, we resort to the class of approximate Bayesian computation algorithms. To select the best algorithm for our problem, we have compared different ABC methods on a simpler analytically tractable drift-diffusion SDE model, referred to as the “toy problem”. Comparing ABC methods directly on the cell movement model is practically infeasible due to its computational complexity.

The drift term in the SDE of the collective cell movement model is derived by considering receptor-ligand kinetics. By looking at the rate at which ligands bind on and off the cell receptors, assuming a constant total receptor concentration, we arrive at the chemotactic term given by (7). This term allows cells to chemotax proportionally to the chemical gradient when the concentration is small relative to the disassociation constant $K_d$, and it induces random cell movement when the concentration is large relative to the disassociation constant. The evolution of the chemoattractant is described using the diffusion equation with constant diffusion coefficient, along with a Gaussian-like term that models the degradation of the chemical by the cells. The strength of the cell degradation is assumed to have a Michaelis-Menten form.

To numerically simulate the movement of the cells and the evolution of the chemoattractant, we have retrieved experimental quantities for the self-generated gradient data from Tweedy et al. (2016) and values for the model parameters from the literature. To simulate the movement of the cells, we solve our drift-diffusion SDE numerically by the Euler-Maruyama method. The evolution of the chemoattractant is simulated on a uniform background grid using an implicit-explicit finite difference scheme to numerically solve (9). The updated concentration is then found by solving a tri-diagonal system of equations. Linear interpolation is used to estimate the chemical concentration at the location of the cells, as well as using a linear approximation for the chemical gradient at the location of the cells. Our simulations have shown a leading wave of cells, a key property of self-generated gradient, and concentration profiles which matched that found in Tweedy et al. (2016). This demonstrates that our drift-diffusion model has the flexibility to give rise to self-generated gradient chemotaxis.

We have compared different ABC approaches to inferring the parameters of the synthetic benchmark problem, the diffusion coefficient *D* and the drift velocity $\alpha$. The adopted model specification renders the exact posterior tractable, allowing us to compare results from ABC algorithms against the known gold-standard. We have investigated the performance of five popular ABC algorithms: rejection ABC, ABC-SMC, ABC-SMC with adaptive distance function of Prangle (2017), semi-automatic ABC of Fearnhead and Prangle (2012), and GP-enhanced semi-automatic ABC of Borowska et al. (2021). To further improve upon the obtained results, we have proposed two modified algorithms: an extension of the GP-ABC algorithm utilising convex hulls, and a two-stage semi-automatic approach, in which a GP regression model is fitted to the residuals from a first stage linear regression model. In all our experiments we have used the MSD calculated at $N=100$ discretisation points as the first-stage summary statistics; for semi-automatic ABC algorithms we then used estimates of the posterior mean for *D* and $\alpha$ as the second-stage summary statistics.

The main finding from our synthetic benchmark study is that regardless of the algorithm used, the ABC-posterior distributions depend crucially on the value of the measurement time interval *T*, as does the exact posterior distribution. For small values of *T*, the exact posterior distribution is relatively tight in *D* and considerably dispersed in $\alpha$, meaning that $\alpha$ is hard to identify. In contrast, for large values of *T*, the exact posterior distribution is more dispersed in *D* compared with $\alpha$, which may lead to identification problems for *D*. However, there are intermediate values of *T* for which the exact posterior distribution is more isotropic, which facilitates identification and precise inference simultaneously for *D* and $\alpha$. These findings mirror the results reported by Devlin et al. (2019), who show that an intermediate value of *T* balances the accuracy of the inference of both parameter values.

Compared to standard approaches based on first-stage summary statistics, the semi-automatic algorithms considered, including the two new ones proposed in this paper, provide more accurate inference of the location of the posterior distribution. Moreover, these methods generally do a (relatively) good job of estimating the uncertainty of *D*, in contrast to the basic methods, such as rejection ABC or ABC-SMC, which tend to produce considerably overdispersed ABC-posterior distributions. We have therefore used the proposed residual ABC approach for inferring *D* and $\nu$ of the cell movement model. As expected, both parameters are inferred relatively accurately.

One prominent challenge, universal for most ABC methods, relates to the use of summary statistics. Our results demonstrate that the semi-automatic approaches, in which second-stage summary statistics are obtained as predictions from a pre-fitted regression model, provide an advantage over standard algorithms based on first-stage summary statistics. However, the semi-automatic methods still rely on manually selected and constructed transformations of the data (first-stage summary statistics). ABC methods without summary statistics seem a promising strand of research (Sousa et al. 2009). More recently, applying the Wasserstein distance to directly convert high dimensional data into a one-dimensional distance value has been proposed (Bernton et al. 2019). However, even such seemingly fully automatic methods still require user inputs, e.g. as to what mapping from the data space to the distance space to use. In other words, application-specific intuition behind selecting summary statistics is traded for intuition behind selecting an appropriate space filling curve.

We have used the MSD as first-stage summary statistics either directly in rejection ABC or ABC-SMC, or indirectly in semi-automatic algorithms, where it served as the explanatory variable in regression models. We note that the 100 time points at which we calculate MSD are uniformly spread over the time domain [0, *T*]. This might be sub-optimal, especially given the relationship between the MSD and the two parameters of interest. Generally, the quadratic nature of the MSD means for small values of *T*, the quadratic term in formula (21) essentially disappears so we cannot estimate $\alpha$ well; on the other hand, for large values of *T*, the relative weight of the linear term becomes negligible so that *D* becomes hard to estimate accurately. Selecting MSDs uniformly intensifies this issue. Such insights can inspire better choices and designs of summary statistics, but that requires domain knowledge that may not always be available.

## Figures and Tables

**Fig. 12:**
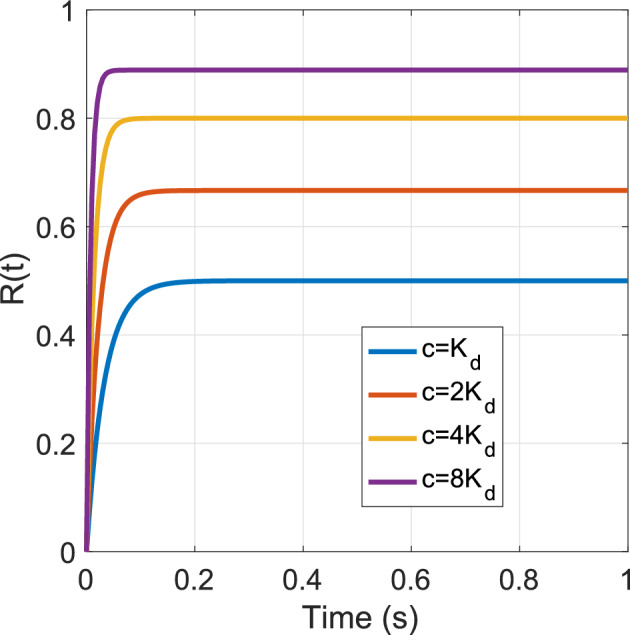
Kinetics of ligand-receptor binding

**Algorithm 2:**
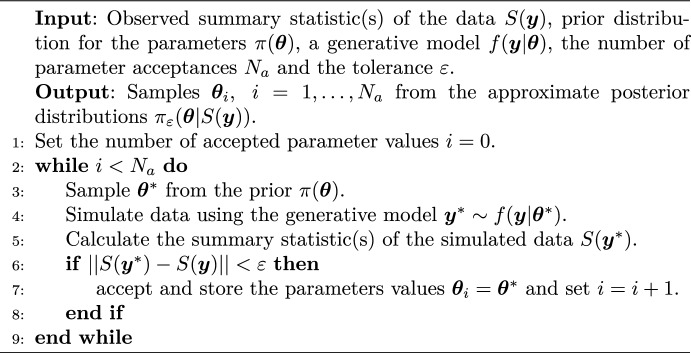
ABC rejection algorithm (Pritchard et al. 1999)

**Algorithm 3:**
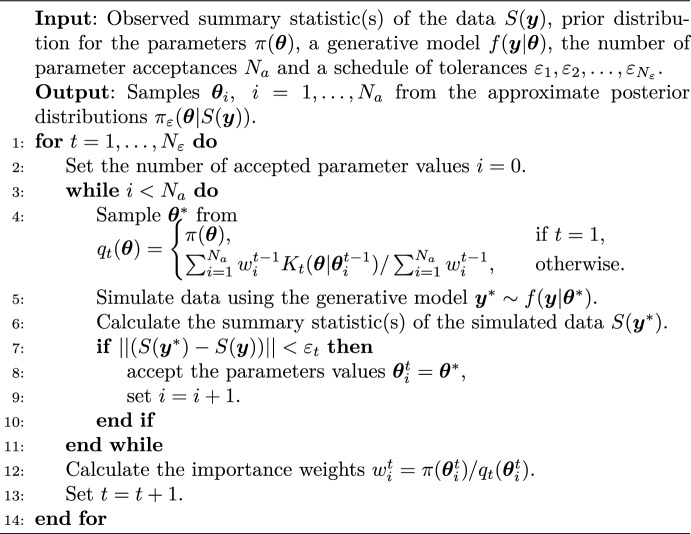
ABC-SMC algorithm (Beaumont 2010)

**Algorithm 4:**
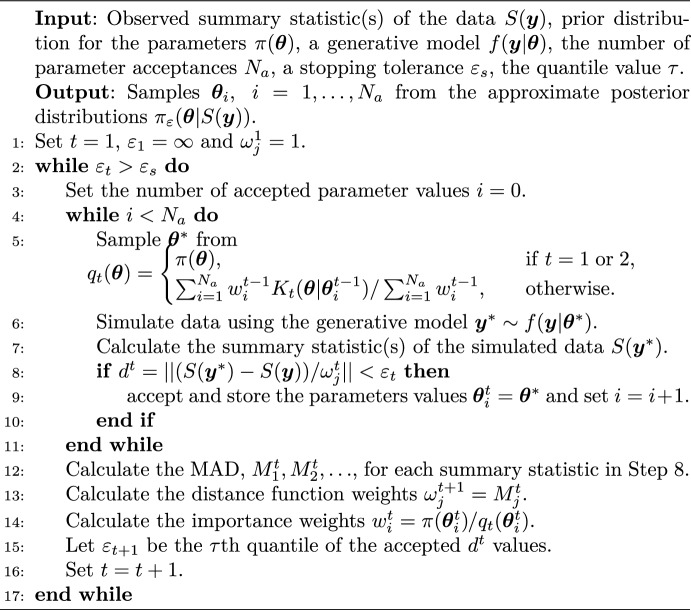
ABC-SMC with adaptive weighting (Prangle 2017)

**Fig. 13:**
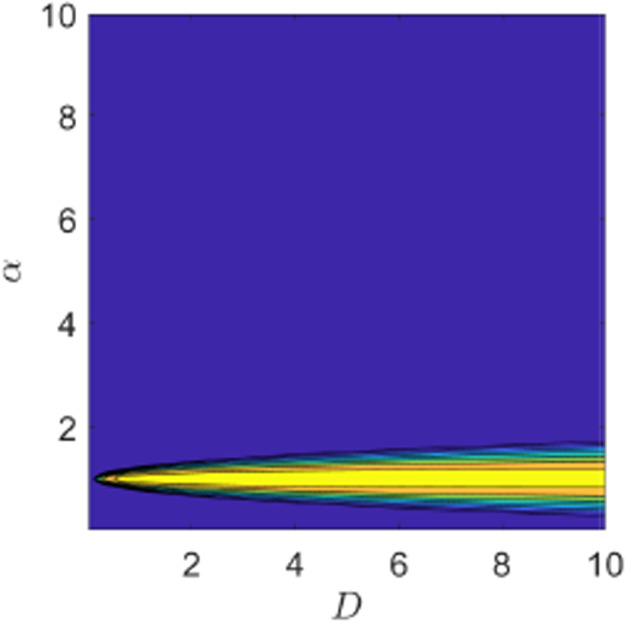
Contour plots of the exact posterior distribution for $T=50,000\, \textrm{s}$

**Fig. 20:**
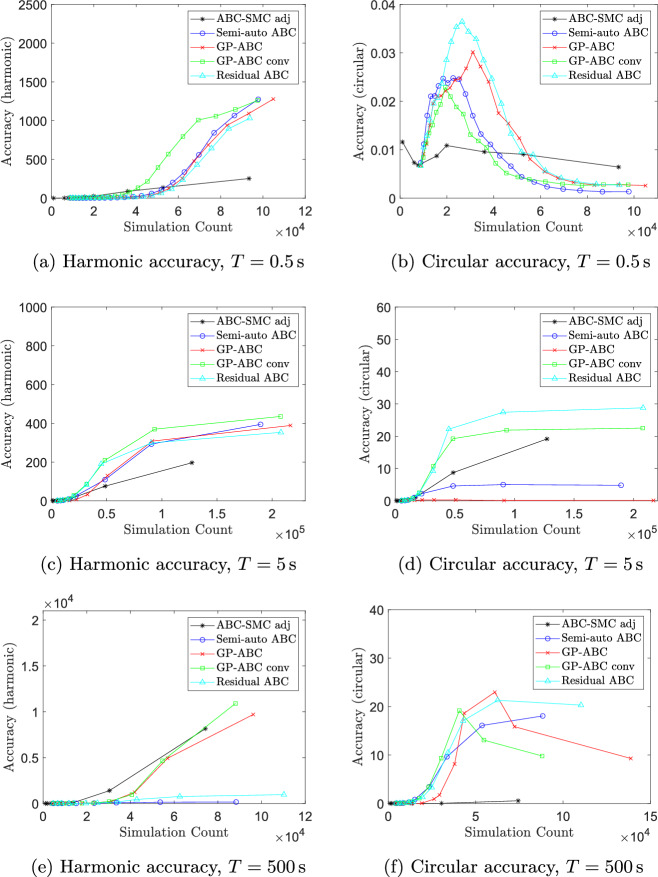
Accuracies against the simulation count for ABC-SMC with adaptive weights (black line with asterisks), semi-automatic ABC with MVLR (blue line with circles), GP-ABC (red line with crosses), GP-ABC with convex hulls (green line with squares) and residual ABC (cyan line with triangles) for the three different values of *T*

## Appendix A: Kinetics of receptor-ligand binding

In this section we consider receptor-ligand binding dynamics to justify the assumption that these occur quickly compared to the other physical processes involved in cell motility. In Fig. 12 we have plotted the evolution of fractional receptor occupancy $R(t)=\psi (t)/R_{tot}$, where $\psi (t)$ is the number of occupied receptors and $R_{tot}$ is the total (occupied and and unoccupied) number of receptors. We have assumed initially that all receptors are unoccupied, the ligand-receptor disassociation parameter $K_{d}=150 \textrm{nM}$ (Wurster and Butz 1980) and the ligand-receptor on rate $k_{1}=0.1 s^{-1}$. For a range of concentrations *c*, we can see that the fractional receptor occupancy rapidly increases to its equilibrium value $R=c/(K_{d}+c)$, and the time to reach equilibrium decreases with increasing concentration *c*. In all situations, equilibrium is reached rapidly and hence this justifies the use of equilibrium conditions for the fractional receptor occupancy term in the modelling of chemotaxis in the SDE (1).

## Appendix C: Additional results

### C.1 Exact posterior for a very large *T* value

Figure 13 illustrates the shape of the exact posterior distribution for a value of *T* much higher than in our simulations in Sect. 4.3.1 and equal to $50,000\, \textrm{s}$. Notice that the marginal for $\alpha$ becomes concentrated around the true value of $\alpha =1$, however the marginal for *D* becomes essentially diffuse and is not centred around the true value $D=2$. One of the reasons for this practical unidentifiability of *D* is related to keeping the number of discretisation points *N* fixed (which is dictated by keeping the computational costs equal between different simulation configurations). However, as investigated by Michalet (2010) (see Fig. 4 there), for diffusion processes increasing the number of fitting points helps to infer diffusion coefficients to a certain point, beyond which it becomes detrimental to use a higher number of fitting points.

### C.2 The impact of the tolerance value on rejection ABC and ABC-SMC

#### C.2.1 Rejection ABC

Figures 14, 15 and 16 show samples from the joint posterior distributions for $T=0.05\, \textrm{s}$, $5\, \textrm{s}$ and $500\, \textrm{s}$, respectively. As we decrease $\varepsilon$, the joint posterior distributions get slowly closer to the exact posterior distributions for all values of *T*. We see a larger improvement for a small and middle value of *T*, while for a large value of *T*, the joint posterior distribution in *D* covers the entire prior distribution.

#### C.2.2 ABC SMC

See Figs. 17, 18 and 19.

## Appendix D: Accuracy measures

As discussed in Sect. 4.3.4, for models with practical identification problems, such as SDE (14), one may be interested in assessing how well an ABC algorithm practically identifies each parameter, or both parameters simultaneously. To this end, we need to define accuracy measures that take the ground-true values of parameters into account. In this Section, we first introduce two such measures, based on mean squared errors (MSEs), and then we use them to compare ABC algorithms. We focus on the five ABC algorithms that we have demonstrated to perform relatively well (see Sect. 4.3.1), i.e. ABC-SMC with adaptive weights (Prangle 2017) and four semi-automatic schemes (based on MVLR, GP regression, GP regression with convex hulls, and the residual approach).

### D.1 Definitions

#### Harmonic accuracy

We first introduce the harmonic accuracy that is predominately determined by the change in the strongly (practically) identifiable parameter (which parameter it is depends on the value of *T*). To define it, we calculate the posterior mean for *D* and $\alpha$ for the *i*th tolerance value, denoted by $\overline{D}_i$ and $\overline{\alpha }_i$, respectively, as well as the variance of the accepted values of *D* and $\alpha$, denoted by $(\sigma _D^2)_i$ and $(\sigma _{\alpha }^2)_i$, respectively. The MSE for *D* and $\alpha$ for the *i*th tolerance value is then given by

$$\begin{aligned} (MSE_D)_i&=(D-\overline{D}_i)^2+(\sigma _D^2)_i, \quad i=1,\ldots , N_{\varepsilon },\\ (MSE_{\alpha })_i&=(\alpha -\overline{\alpha }_i)^2+(\sigma _{\alpha }^2)_i, \quad i=1,\ldots , N_{\varepsilon }, \end{aligned}$$

where *D* and $\alpha$ are the true parameter values, and $N_{\varepsilon }$ is the number of tolerance values. The harmonic accuracy for the *i*th tolerance value is then given by

31 $$\begin{aligned} \mathcal {A}^{\mathcal {H}}_i=\frac{1}{(MSE_D)_i}+\frac{1}{(MSE_{\alpha })_i}, \quad i=1,\ldots , N_{\varepsilon }. \end{aligned}$$

#### Circular accuracy

We will define the second accuracy measure so that a higher level of accuracy corresponds with estimating both parameters well, rather than only the strongly (practically) identifiable parameter. To do this, we need to think about what it means for both parameters to be estimated well. In two dimensions, we could think of this as the shape of the level sets of joint posterior distribution being circular/elliptical around the true parameter values an appropriately small radius.

To implement this idea, we need a measure of circularity of a general shape. One simple measure would be the compactness measure [?], defined as $C~\!=~\!4\pi A/p^2$, where *A* is the area enclosed and *p* is the perimeter of the shape. If the shape is a circle then $C=1$ and $C<1$ otherwise.

For posteriors roughly rectangular in level set plots, we set $A=MSE_D \times MSE_{\alpha }$ and $p=2(MSE_D+MSE_{\alpha })$. This gives an approximate value of $C \approx \pi ( MSE_D \times MSE_{\alpha })/(MSE_D+MSE_{\alpha })^2$. Since *C* is maximised when the area is close to a circle, dividing by *C* puts more emphasis on estimating both parameters well, rather than just one of them. A possible error measure could then be specified as $\sqrt{A}/C \approx (MSE_D+MSE_{\alpha })^2/\sqrt{MSE_D \times MSE_{\alpha }}$. We then define our second accuracy measure, which we call the circular accuracy, as the inverse of the error, giving

32 $$\begin{aligned} \mathcal {A}^{\mathcal {C}}_i=\frac{\sqrt{(MSE_D)_i \times (MSE_{\alpha })_i}}{((MSE_D)_i+(MSE_{\alpha })_i)^2}, \quad i=1,\ldots , N_{\varepsilon }. \end{aligned}$$

Note that this measure of accuracy could be extended to higher dimensional parameter spaces by using higher-dimensional analogues for the area and perimeter.

### D.2 Results

A comparison of the harmonic and circular accuracy against the simulation count for ABC-SMC with adaptive weights and the four semi-automatic ABC algorithms (semi-automatic ABC with MVLR, GP-ABC, GP-ABC with convex hulls, and the residual approach) is given in Fig. 20.

#### Small *T*

For $T=0.05\, \textrm{s}$, the harmonic accuracy for all the semi-automatic approaches is comparable, with GP-ABC with convex hulls achieving the highest value the quickest. The semi-automatic approaches considerably outperform ABC-SMC with adaptive weight, which corresponds to the narrower ABC-posterior distribution in the practically identifiable parameter *D* for the former compared to the latter (see Fig. 5).

As for the circular accuracy, for all the algorithms it initially increase as both parameters are inferred more accurately, but then falls as the joint posterior distributions remain wide in $\alpha$. ABC-SMC with adaptive weights performs best in this case, as the corresponding ABC-posterior distribution is most circular. Semi-automatic ABC with MVLR results in the lowest circular accuracy, which is due the ABC-posterior of $\alpha$ being the most dispersed among those for the four semi-automatic algorithms (see Table 4).

#### Medium *T*

For $T=5\, \textrm{s}$, the harmonic accuracy exhibits a similar patter as for $T=0.05\, \textrm{s}$, with GP-ABC with convex hulls performing the best and ABC-SMC with adaptive weight performing worst.

This time, however, the circular accuracy monotonically increases in the simulation count, which corresponds to the ABC-posterior distributions becoming more and more isotropic. The residual approach results in the highest circular accuracy, with GP-ABC with convex hulls coming second. The two standard semi-automatic algorithms perform relatively poor in this case.

#### Large *T*

For $T=500\, \textrm{s}$, GP-ABC with convex hulls again delivers the highest harmonic accuracy, with the standard GP-ABC being second-best. Interestingly, ABC-SMC with adaptive weights works relatively well in this case. This is due to the ABC-posterior for this method being relatively tight in $\alpha$, especially compared with semi-automatic ABC with MVLR (also see Table 4).

As far as the circular accuracy is concerned, the semi-automatic approaches clearly outperform ABC-SMC with adaptive weights. Among the former, the residual approach results in the highest circular accuracy overall, which reflects the most isotropic ABC-posterior distribution for this algorithm.

#### Conclusions

We find that the two semi-automatic schemes proposed in this paper (Sect. 3.4) result in the highest accuracy measures for the three values of *T*. When it comes to accurately inferring the practically identifiable parameter, GP-ABC with convex hulls performs best, as quantified by the highest values of the harmonic accuracy for this algorithm. As for inferring both parameters simultaneously, the residual approach is generally superior, with the largest circular accuracy values (except for long simulations for $T=0.05\, \textrm{s}$, where it becomes overtaken by the ABC-SMC algorithms with adaptive weights).

## Footnotes

1 For the sake of generality, we will refer to any entity which moves as a particle.

2 The triangular distribution is described in Evans et al.  (2000) and is characterised by three values (*a*; *b*; *c*), where *b* is the nominal value of the parameter, and *a* and *c* are the minimum and maximum values, respectively
